# Molecular Dynamics Simulations Suggest That Side-Chain Motions of Charged Amino Acids Determine Long-Range Effects in Proteins: An Egg of Coulomb

**DOI:** 10.3390/ijms252413375

**Published:** 2024-12-13

**Authors:** Neri Niccolai, Edoardo Morandi, Andrea Bernini

**Affiliations:** Department of Biotechnology, Chemistry and Pharmacy, University of Siena, 53100 Siena, Italy; andrea.bernini@unisi.it (A.B.)

**Keywords:** protein–ligand interactions, protein long-range communications, protein surface electrodynamics

## Abstract

Living systems cannot rely on random intermolecular approaches toward cell crowding, and hidden mechanisms must be present to favor only those molecular interactions required explicitly by the biological function. Electromagnetic messaging among proteins is proposed from the observation that charged amino acids located on the protein surface are mostly in adjacent sequence positions and/or in spatial proximity. Molecular dynamics (MD) simulations have been used to predict electric charge proximities arising from concerted motions of charged amino acid side chains in two protein model systems, human ubiquitin and the chitinolytic enzyme from *Ostrinia furnacalis*. This choice has been made for their large difference in size and sociality. Protein electrodynamics seems to emerge as the framework for a deeper understanding of the long-distance interactions of proteins with their molecular environment. Our findings will be valuable in orienting the design of proteins with specific recognition patterns.

## 1. Introduction

In the molecular crowding typical of biological systems, protein–protein interactions cannot be driven by fortuitous encounters. Even though this statement is rather apparent, limited advances have been made to understand the mechanisms controlling long-range molecular dialogs among biomolecules at an atomic level. In this respect, Brownian dynamics simulations can offer much information on short-distance biomolecular interactions by considering electrostatic, hydrodynamic, and hydrophobic effects [1,2]. Afterward, transient encounter complexes, proposed as preliminary steps for forming protein–protein complexes [3], can be considered for further in silico screenings. However, how molecular partners are driven to come nearby to establish transient encounters and, eventually, for their complex formation remains largely unknown. Indeed, the brilliant description that Herbert Fröhlich made fifty years ago on the dielectric properties of biomolecules [4,5] was not considered the basis of deciphering the mechanisms of long-range intermolecular interactions. Only in the last decade have several reports appeared, suggesting that resonant long-range interactions between polar macromolecules occur [6,7]. Then, the fact that experimental data eventually confirmed theoretical predictions [8,9] is of primary relevance for such a fundamental aspect of biomolecular recognition.

A structural bioinformatic survey on a sizeable, non-redundant array of proteins, whose Protein Data Bank (PDB) structures ensured the absence of molecular shape alteration due to protein–ligand interactions [10], revealed the presence of many possible electric dipoles distributed on protein surfaces. Indeed, quantitative analysis of the amino acid composition of the most external layer of the aforementioned selected proteins revealed that lysines and glutamates resulted in being, by far, the most abundant amino acids, with occurrences of 17% and 15%, respectively. This finding is not surprising, considering the role of charged surface moieties in ensuring suitable solubility, structural stability, and intermolecular interactions. However, the fact that the two aforementioned amino acids were found most frequently as sequence neighbors was less expected. Furthermore, DSSP assignments [11] of the protruding EK and KE fragments of the selected proteins indicated their predominant location in helices and, to a lesser extent, in turns, i.e., the two secondary structure elements that allow close approaches between the side chains of adjacent residues. KK and EE dipeptides were also frequently found in the outer layer of protein, sharing the same secondary structure elements observed for EK and KE.

Due to the high flexibility expected of charged amino acid side chains on the protein surface, the resulting electric charge distribution cannot be analyzed simply by inspecting static structures. Hence, MD simulations, offering a reliable reference for investigating time-dependent structural features in proteins [12], are proposed as the computational basis for characterizing the evolution of the protein electric charge network.

Here, protein surface electrodynamics as the engine driving long-range intermolecular interactions is explored by analyzing the distribution and motion of the charged side chains of two protein model systems, which can be well representative of different molecular sizes and interactivity patterns. One of the two protein model systems is human ubiquitin, hUBQ, a well-characterized protein [13] that is considered among the most social ones [14], a behavior that has been ascribed to its high content of charged residues [15]. The other protein model system is a very large chitinolytic enzyme from *Ostrinia furnacalis*, ofCE, which performs its catalytic role without other protein partners [16].

The relevance of our work is twofold. MD simulations indicate how electric local changes at the protein surface are caused by the fast reorientation of charged side chains, which consequently induces long-range electromagnetic effects. Furthermore, this is not the case for charged active site residues, as they must be in a constant electric environment to maintain their biological function. Protein de novo engineering in the artificial intelligence era should consider the present findings and analyses.

## 2. Results

A sequence analysis of hUBQ and ofCE confirms the already-observed frequent recurrence of adjacent residues bearing opposite electric charges [10]. Moreover, hUBQ and ofCE charged dipeptides are predominantly located in secondary structure elements which favor close side-chain proximity. This sequence-based preliminary consideration prompted a systematic structural search for all short-distance interactions among the charged residues of the two proteins. Such an investigation was carried out through MD simulations in explicit water to account for side-chain and backbone flexibility. To simplify all our calculations, the histidyl side chain was considered always positively charged, even though local environments could also modify this electric property.

### 2.1. Charged Side-Chain Dynamics in Human Ubiquitin

**Figure 1:**
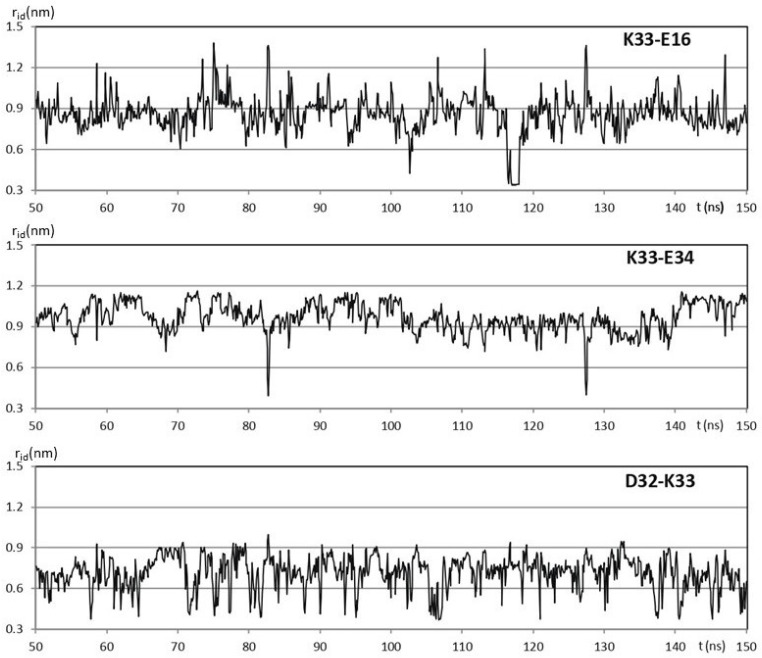
100 ns windows of the 500 ns trajectory obtained for hUBQ MD simulation in explicit water. The time evolution of the K33 NZ distance r_id_ with 16E CD, D32 CG, and E34 CD is shown.

Among the 76 residues of the hUBQ sequence, there are 23 charged side chains, 5 D, 6 E, 1H, 7 K, and 4 R, mostly surface-exposed, as consistently found in all the solution and crystal ubiquitin structures that are available in the PDB [17]. Along the hUBQ sequence, the KE/EK motif underlined above is present only twice, i.e., K33E34 and K63E64. However, within the protein conformational dynamics, many other short-distance pairwise interactions among the 23 hUBQ charged residues occur. To quantify the aforementioned interactions, the relative positions of lysyl NZ, glutamyl CD, aspartyl CG arginyl CZ, and histidyl CE1 atoms (according to the PDB amino acid nomenclature) were monitored along a 500 ns MD simulation in explicit water to identify short-distance reciprocal approaches among D, E, H, K, and R along the MD trajectory. Thus, we systematically searched for internuclear distances, rid, between the aforementioned atoms that we have defined as probe atoms to characterize the approaches among the charged side chains. It is worth noting that rids shorter than 0.4 nm are consistent with the formation of transient hydrogen bonding between charged side chains also in the case of sequence-distant amino acids. All the short-distance approaches among the electric charges that are present in hUBQ were characterized by sampling r_id_s along the hUBQ MD trajectory with g_distmap, an ad hoc developed GROMACS tool [18]; see the details given in Supplementary Materials. The relevant short-distance interactions among D, E, H, K, and R residues were considered only if they exhibited at least 5% of the r_id_s calculated to be below 0.6 nm. Under this limiting condition, from the total 23 × 23 r_id_ matrix, a reduced set of 32 pairwise interactions was obtained. Figure 1 shows the evolution of r_id_ of hUBQ K33 NZ with 16E CD, D32 CG, and E34 CD during a 100 ns window of the entire 500 ns MD trajectory. Three different conformational arrangements occur for the oppositely charged side chains of K33, D32, and E34, all implying the formation of hydrogen bonding, as confirmed by the r_id_ values lower than 0.4 nm. From the data shown in Figure 1, it is also apparent that the three H bonds that it establishes with its charged side-chain neighbors are very different in terms of duration, frequency of formation, and, most likely, orientation. 

**Figure 2:**
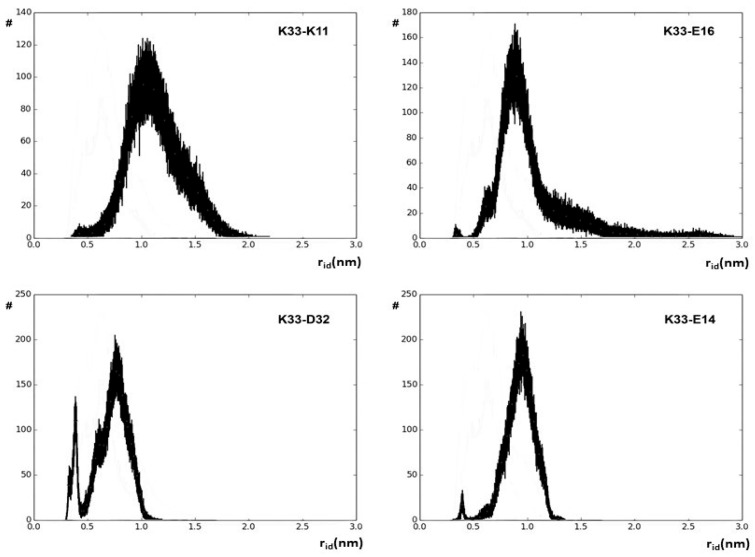
Binned values of r_id_ distances along the entire 500 ns hUBQ MD trajectory delineating the approach’s evolution between the K33 NZ atom and some of the probe atoms of the neighboring charged side chains.

**Figure 3:**
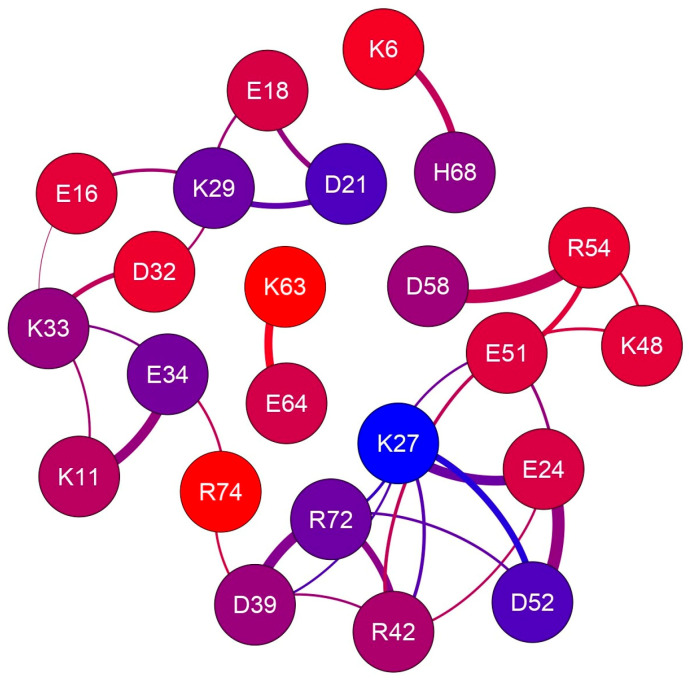
The electric charge interaction network in human ubiquitin. The 23 nodes, representing all charged residues that fulfill our limiting criteria, are colored according to the depth of probe atoms, as reported in Table 1; edge thickness is proportional to the average proximity.

The r_id_ profiles obtained by using the g_distmap tool from GROMACS v2019.3 for K33 NZ, fulfilling the used limiting filter, are shown in Figure 2. The relevance of K33 NZ H bonding with nearby glutamyl and aspartyl side chains can be inferred directly by the number of occurrences in the 0.4 nm region of the aforementioned r_id_ profiles. Figure S2 shows the r_id_ binning profiles that were generated for all the 32 approaching charged side chains that fulfill our limiting conditions, and Figure 3 shows the complete interaction network among hUBQ charged side chains. The fact that all side chains bearing opposite charges establish one or more reciprocal interactions is the first feature that emerges from the data shown in Figure 3, despite sporadic short-distance approaches, e.g., in the case of the most promiscuous K27 side chain. Furthermore, it must be noted that K63, a critical residue in the polyubiquitin chain formation [19], exhibits only a single strong interaction with the nearby E64, ensuring the constant charge distribution and dipole orientation that are required for maintaining its biological role. Similarly, we can also note that D58 establishes only a single electric interaction with R54, stabilizing the hUBQ polar binding domain [20]. 

The time scale of dipole oscillations arising from the side-chain motion of charged amino acids can be estimated from NMR relaxation studies. Indeed, the order parameters, S^2^, of hUBQ lysyl ^15^NZ atoms have been calculated, as well as rotational correlation times accounting for χ_5_ internal side-chain rotations [14], indicating how the various lysyl NZ atoms experience very different internal dynamics. Our MD results are in good agreement with the experimental data from [21], as the highest S^2^ value determined for the buried K27 NZ atom is consistent with the very limited population of conformers where the K27 NZ atom is free from hydrogen bonding with the neighboring E24, D39, E51, and D52 carboxyl acceptors; see Figure S2 (panels 16 and 26). Moreover, the NZ nitrogen of K11, K33, K48, and K63 have the lowest S^2^ values together with high atom depth indexes; see Table 1 and Figure S1. From the same ^15^N NMR relaxation study, lysyl χ_5_ reorientational correlation times were calculated to range from 25 to 341 ps, respectively, for K63 and K27, where the surface-exposed K63 reorients almost fourteen times faster than the inner K27.

### 2.2. Charged Side-Chain Dynamics in the Ostrinia Furnacalis Chitinolytic Enzyme

We have selected the *Ostrinia furnacalis* chitinolytic enzyme, ofCE, as a second protein model system as it contains 151 charged side chains at the amino acid composition (see Figure S3), which is very similar to the average values found in the SwissProt database [22]. Its crystal structure, PDB ID code 3NSM, shows the 572 residues of the enzyme assembled in two domains to form a large globular shape. Then, the same procedure applied to hUBQ was used to investigate the ofCE electric charge network. From the 3NSM structure, we have derived the relative positions of 41 D, 38 E, 9 H, 35 K, and 28 R residues. Most of these 151 residues are mainly distributed on the 592.77 nm^2^ protein surface, which is almost eight times larger than that of hUBQ. The time-dependent spatial proximity among the electric charges of the aforementioned residues was estimated from a 30 ns MD simulation in explicit solvent. By applying to ofCE the same limiting conditions used for hUBQ, we delineated 209 short-distance approaches among the charged side chains with rid profiles that are reported in Figure S4. Figure S5 shows examples of the E34 CD-K11 NZ, D58 CG-R54 CZ, and H244 CE1-E297 CD pairs that involve the formation of inter-residue hydrogen bonds. Table S1 reports atom depth indexes and DSSP assignments for all the aforementioned lysyl NZ, glutamyl CD, aspartyl CG, histidyl CE1, and arginyl CZ atoms of ofCE. Differently from hUBQ, the ofCE interaction network presents charged residues that are not involved in any short-distance interactions with other charges. Among these residues, listed with null values of the interaction ranking reported in Table S1, it is interesting to note that K323 is directly involved in binding with NAG and TMG-chitotriomycin in the enzyme–inhibitor complex (PDB ID code: 3VTR), suggesting that unperturbed charge density is required by protein hot spots. The other 143 charged residues are involved in 203 side-chain–side-chain interactions with ranking values of up to 7 for the most social cases offered by the deeply buried H246 and H303 residues; see Table S1.

**Figure 4:**
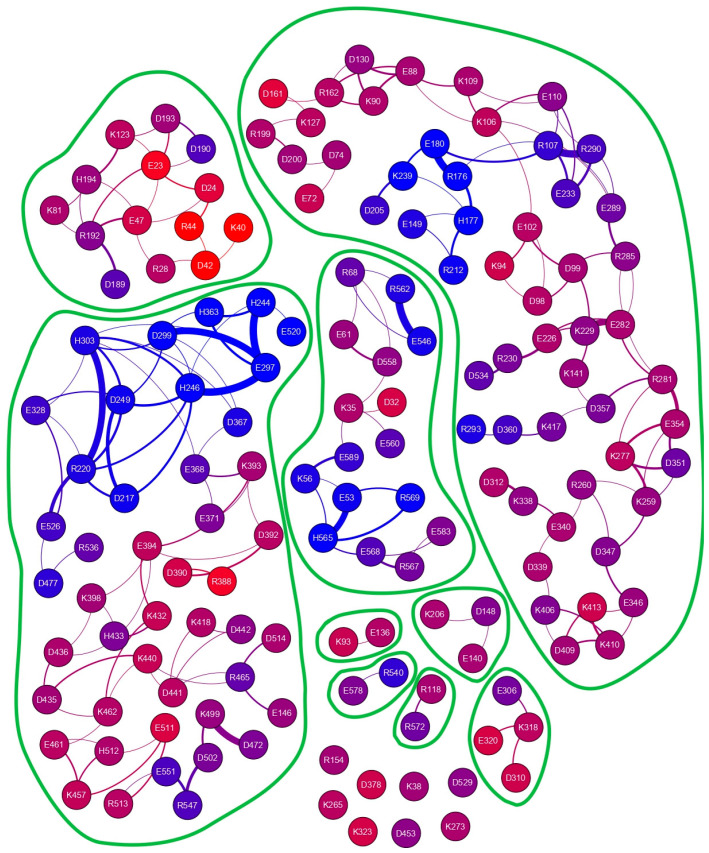
Charged side-chain interaction network of the Ostrinia furnacalis chitinolytic enzyme. Different interaction patches can be identified. The 151 nodes, representing all the charged residues that fulfill our limiting criteria, are colored according to atom depths obtained with a probing sphere of 1.2 nm, as reported in Table S1, which summarizes some of the parameters underneath the graph.

Similarly to what we have found for hUBQ, close-distance approaches among charged side chains are mainly due to opposite charges—137 (67.6%)—but in addition, 33 positive-to-positive and negative-to-negative encounters (16.2% each) satisfy our limiting conditions for selecting short-distance interactions. As shown in Figure 4, the ofCE charged side chains exhibiting close-distance approaches are grouped into different patches, defined by the absence of edges in the interaction network that identify the boundaries of their concerted motions.

## 3. Discussion

**Figure 5:**
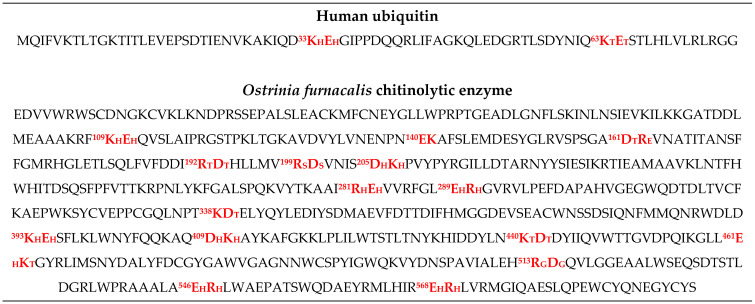
The hUBQ and ofCE dipeptide fragments with charged amino acids are highlighted in red; the numbers refer to the residue positions, and the subscripts define DSSP assignments, as reported in the caption of Table 1.

**Figure 6:**
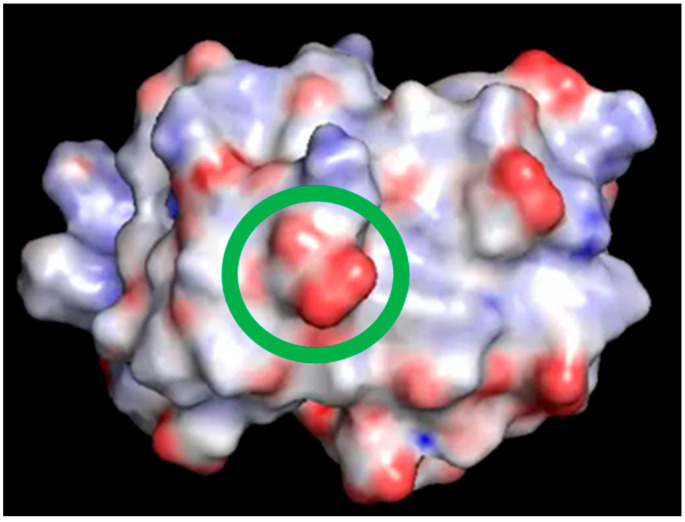
One frame of the video included in Supplementary Materials. The surface of hUBQ was colored using the Pymol electrostatics plug-in APBS; the green circle highlights D58 carboxylic moiety.

The existence of experimental evidence that proteins possess electrodynamic properties [4,5,6,7,8,9] has prompted the present investigation on the distribution and motion of electric charges in amino acid side chains. We have noticed, indeed, that charged amino acids with the most flexible side chains, i.e., lysine and glutamate, are frequently found in contiguous sequence positions. As shown in Figure 5, this peculiar feature holds in the case of hUBQ and CE, two proteins that are very different in terms of size and biological activity. The structural information offered by 1UBQ and 3NSM PDB files indicate that EK and KE fragments are located in secondary structure elements such as helices and turns that make their reciprocal interactions possible. All the dipeptide fragments composed of oppositely charged side chains also exhibit similar behavior, suggesting that protein sequences and tridimensional structures favor close encounters among electrically charged side chains, which we have computationally verified with the above-described MD simulations. Furthermore, the interaction networks shown in Figure 2 and Figure 4 delineate the different behaviors of each charged amino acid, as some of them are not involved in close approaches with the other ones or are just in a single pairwise interaction. In hUBQ, this is the case for D58 and K63, two residues that are, respectively, critical for establishing the protein’s polar binding domain and to favor polyubiquination. Figure 6 shows one of the most interesting features that is observed through a video, given in Supplementary Materials as hUBQ.wmv. Indeed, the video, generated by the MD simulation trajectory, clearly shows the persistence of the negative charge and orientation of the D58 side chain, critical for protein binding. Similar constant behavior in terms of negative electric charge distribution and side-chain orientation is exhibited by K63, in contrast with most of the remaining charged protein moieties that continuously change the electric properties of the hUBQ surface. A preliminary deduction can be drawn from these findings: active site residues must be located in a constant electric environment to maintain their biological functions. At the same time, the extensive variability of local electric density, due to multiple charge-to-charge approaches (see Figure 3 and the hUBQ video) is needed for labeling the protein based on its unique composition and structure. 

**Figure 7:**
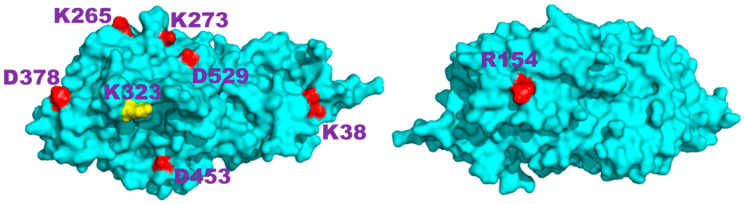
Front and back views of the structure of the Ostrinia furnacalis chitinolytic enzyme obtained from the 3NSM PDB entry; the eight residues that do not have other electric charges nearby are highlighted; K323, positioned at the rim of the catalytic site, is colored in yellow.

Analyzing the complexity in the charge interaction network obtained for ofCE, we have found eight residues that exhibit no charge-to-charge interactions. These residues without other neighboring charged residues include K323, which is located at the rim of the deep enzyme active site. This finding seems to assign to K323 the role of beacon to favor the ofCE substrate recruitment with its lone positive charge. As shown in Figure 7, all eight electric charges are exposed to the enzyme surface and, hence, can freely reorient without affecting local electric properties. This feature yields an electric fingerprint of the ofCE surface overlapping with the electrodynamic effect arising from the mutual interaction among the other 143 charged side chains. The close-distance mutual interactions, summarized in Figure 4, generate 203 electric dipoles originating electromagnetic waves that differ in terms of lifetime, intensity, and frequency. In the case of hUBQ, the ^15^N nuclear relaxation data that we have described above indicate how the time scale of lysyl mobilities is in the terahertz range with large variations between the slow inner K27 and the fast outer K63.

Thus, lone electric charges are often located on the protein surface to accomplish specific functional duties, such as the above-mentioned ones, or to yield a constant electric pattern for short-distance protein recognition. On the other hand, the large array of hUBQ and ofCE electric dipoles that continuously fluctuate in intensity and orientation are free from functional duties, apart from maintaining structural stability and a suitable interaction with solvent molecules. In this respect, it is also interesting to note that the different social behaviors exhibited by hUBQ and ofCE correlate very well with those of the much larger concentration of charged side chains that is present in the small human protein than in the other one. It must be noted that the broad absorption spectrum in the microwave region of water molecules [23] in the biological milieu should not interfere with the electromagnetic signature of proteins. Terahertz spectroscopy [24,25], with systematic investigations on the functional effects of charged amino acid substitutions to specific interacting protein systems, e.g., with E/Q and D/N replacements, is a promising experimental approach for supporting our hypothesis that long-distance protein–protein communications arise from electromagnetic effects. Surface Plasmon Resonance [26] could be also a powerful technique for delineating the effects of modified charge networks in protein–protein interactions by analyzing the k_on_ rate constants of suitable mutants.

## 4. Materials and Methods

MD simulations of 500 ns and 30 ns, respectively, for hUQB and ofCE were performed by using the GROMACS package v2019.3 [18] on 1UBQ and 3NSM PDB structures, solvated in a cubic box of equilibrated TIP3P water molecules [27]. The time-dependent evolution along the MD trajectories of all the approaches among charged side chains was monitored by calculating in each of the MD frames, with a sampling interval of 1 ps, the distance, rid, between lysyl NZ, glutamyl CD, aspartyl CG arginyl CZ, and histidyl CE1 atoms. These atoms were selected as representative ones to assign electric charge positions irrespective of the actual electric charge distribution. Upon binning rid distances determined along the MD trajectory, all the possible combinations among the aforementioned atoms were analyzed with an ad hoc GROMACS tool, g_distmap, which had two groups of atoms as input. Group 1 included atoms A, B, C, and D, and group 2 included atoms E, F, and G. g_distmap calculated, along the MD trajectory, all the distances, i.e., AE, AF, AG, BE, BF, etc. For each atom pair, an n-dimensional XVG file could be obtained where the distances and simulation times are coupled. Then, only side-chain interactions having a number of distances longer than 5% of rids that were < 0.6 nm were selected for further analysis. The interaction networks among the charged side chains were analyzed and visualized by using Gephi 0.8.2-beta software [28]. PyMOL v 1.7 was used for all molecular graphics and, through the APBS plug-in [29], for electrostatics calculations on each MD simulation frame. Atom depth indexes, D_i_, were calculated by using the SADIC algorithm v1.1, as explained in Supplementary Materials.

## 5. Conclusions

Direct evidence of the electromagnetic effects that we have predicted from MD simulation analyses is experimentally difficult to analyze. However, the presence of charged amino acid side chains exhibiting fluctuating short reciprocal distances and very fast reorientations, particularly in the case they are on protein surfaces, must generate electromagnetic patterns that make proteins long-distance-recognizable in the crowded environment of the cell. As a final remark, it should be underlined that, with the increasing amount of de novo engineered proteins with specified intermolecular-interaction properties developed under the assistance of artificial intelligence procedures [30], the protein network of electric charges, the egg of Coulomb, that we have described in the present report should be carefully considered.

## Figures and Tables

**Table 1:** Number of interactions, depth index and DSSP assignment (E = extended, S = bend, H = helix) for selected atoms.

Atom	No. of Interactions	D_i,9_^a^	DSSP ^b^
NZ K6	1	1.29	E
NZ K11	2	1.06	N/A
CD E16	2	1.22	E
CD E18	2	1.16	N/A
CG D21	2	0.60	S
CD E24	3	1.17	H
NZ K27	6	0.35	H
NZ K29	4	0.77	H
CG D32	2	1.23	H
NZ K33	4	0.93	H
CD E34	3	0.76	H

^a^ Depth index calculated at 9 Angstrom; ^b^ E = extended, S = bend, H = helix, N/A = not available.

## Data Availability

Data is contained within the article and Supplementary Materials.

## Supplementary Materials

The following supporting information can be downloaded at https://www.mdpi.com/article/10.3390/ijms252413375/s1.
