# Supplementary material for: Molecular Dynamics Simulations Suggest That Side-Chain Motions of Charged Amino Acids Determine Long-Range Effects in Proteins: An Egg of Coulomb
Source: Int J Mol Sci. 2024 Dec 13;25(24):13375. doi: 10.3390/ijms252413375 (PMC12381767; doi:10.3390/ijms252413375)
Supplement: Supplementary file 1 [file ijms-25-13375-s001.zip › ijms-3270597-supplementary.pdf]

Supplementary material

Long-range electromagnetic effects can drive protein-protein approaches: an egg of Coulomb

Neri Niccolai, Edoardo Morandi and Andrea Bernini

Department of Biotechnology, Chemistry and Pharmacy, University of Siena, 53100 Siena, Italy.

Atom depth calculations

Atom depth is considered among the structural descriptors that correlate protein structure with folding and functional properties. The distance between an atom and the nearest water molecule or the closest surface dot has been proposed as a measure of the atom depth, but, in both cases, the three-dimensional character of depth is lost. To account for the overall molecular shape in atom depth calculations, measurements of intersections between the molecular volume and a sampling sphere of suitable radius, centered on the atom whose depth has to be quantified, have been proposed by the authors in a previous paper [1]. It is apparent that the smaller the exposed volume, the deeper the three-dimensional insertion of the investigated atom inside the molecular structure.

As the atom depth parameter depends on the overall size and shape of the object under discussion, atom depth indexes, D_i's are suggested for characterizing atom insertions within the considered molecular systems. Thus, for an atom i of a given molecule and a sampling radius r, a depth index $D_{i,r}$ may be defined as

$$D_{i,r} = 2V_{i,r}/V_{0,r}$$

where $V_{i,r}$ is the exposed volume of a sphere of radius r centered on atom i and $V_{0,r}$ is the exposed volume of the same sphere when centered on an isolated atom. Thus, the limiting D_i values 0 and 2 are obtained for a totally buried or totally isolated atom, respectively.

---

[1] D. Varrazzo, A. Bernini, O. Spiga, A. Ciutti, S. Chiellini, V. Venditti, L. Bracci, N. Niccolai, Three-dimensional computation of atom depth in complex molecular structures, Bioinformatics 21 (2005) 2856-2860.

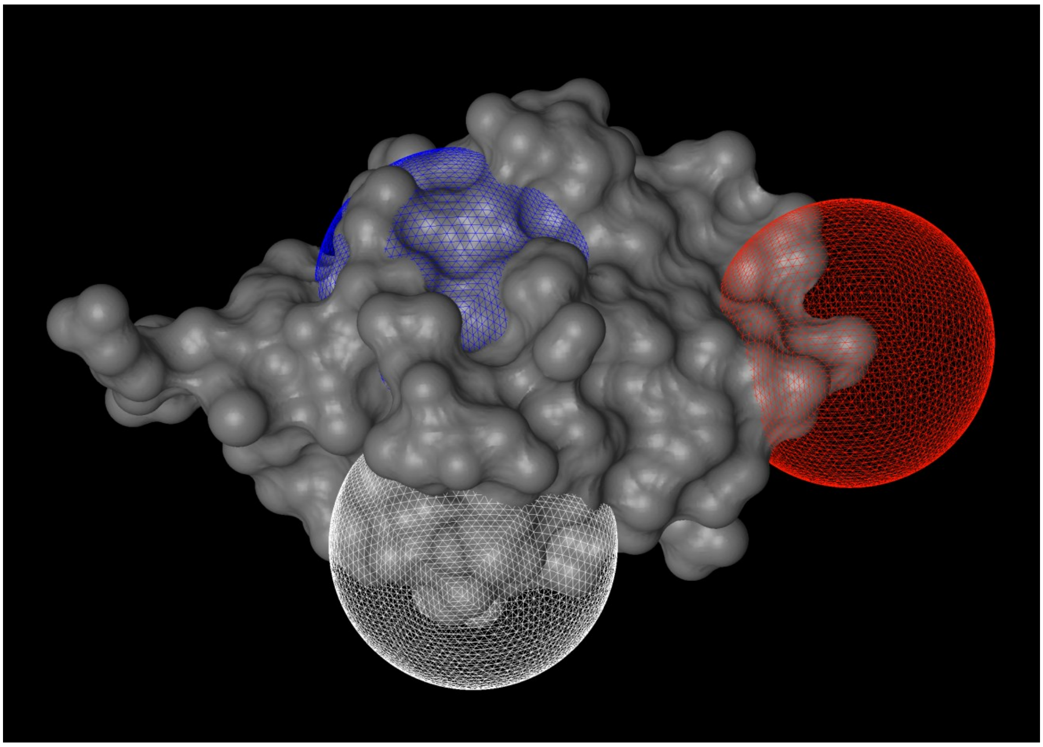

Figure S1: The exposed volumes of L43 CA (buried, color blue, $D_{i,9}$ = 0.05), E34 CD (intermediate, color white, $D_{i,9}$ = 0.76), K63 NZ (fully exposed, color red, $D_{i,9}$ = 1.44) are shown as mesh on the human ubiquitin surface (PDB: 1UBQ) by drawing a sphere of 9 Å radius on each atom.

The algorithm for the calculation of D_i, extensively described in [1], has been implemented in the software Simple Atom Depth Index Calculator (SADIC), whose complete documentation is available at https://sadic.sourceforge.net/. The software outputs the D_i values calculated for the protein structure given in PDB format in the B-factor field of the same PDB file, allowing for coloring by depth index with the most common graphical viewers, like PyMol (http://www.pymol.org).

Figure S2: *Binned distributions of close distance interactions between D, E, H, K, and R side chains in hUBQ as calculated along the 500 ns MD trajectory with g_distmap tool. Only side chain-side chain approaches exhibiting more than 5 % occurrences at r_{IC} < 0.6 nm have been considered.*

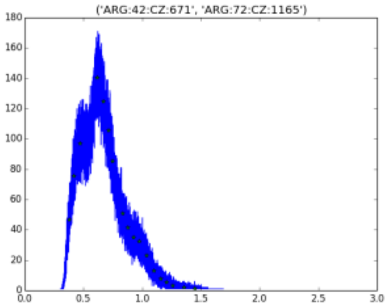

1. R42 CZ – R72 CZ

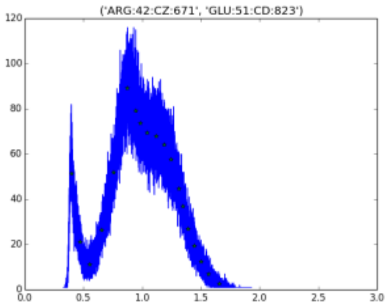

2. R42 CZ – E51 CD

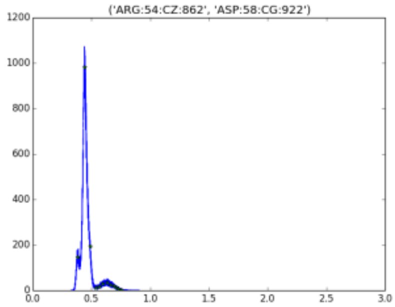

3. R54 CZ – D58 CG

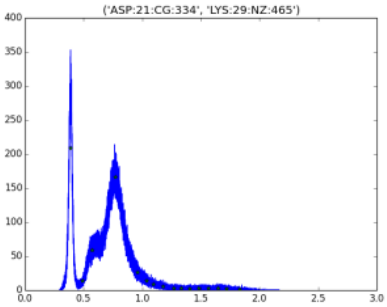

4. D21 CG – K29 NZ

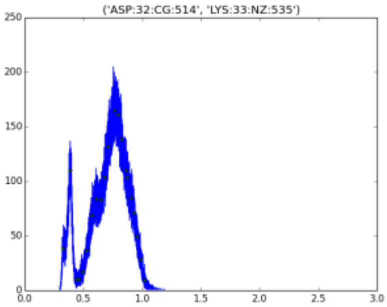

5. D32 CG – K33 NZ

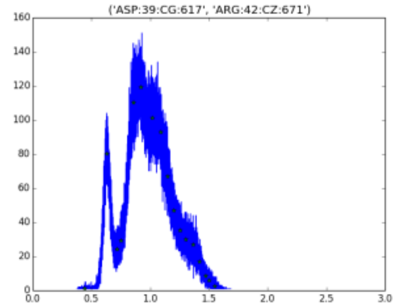

6. D39 CG – R42 CZ

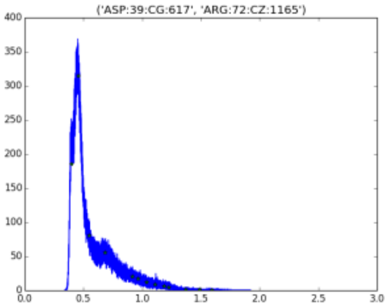

7. D39 CG – R72 CZ

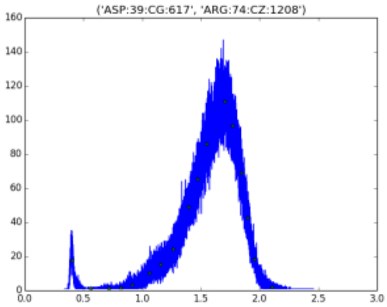

8. D39 CG – R74 CZ

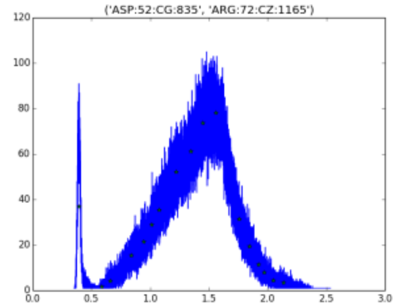

9. D52 CG – R72 CZ

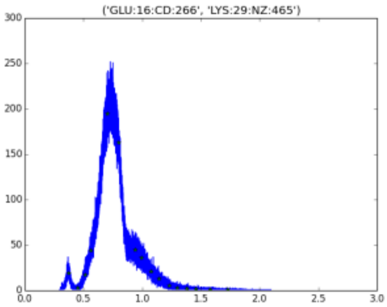

10. E16 CD – K29 NZ

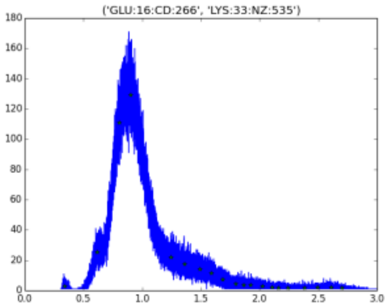

11. E16 CD – K33 NZ

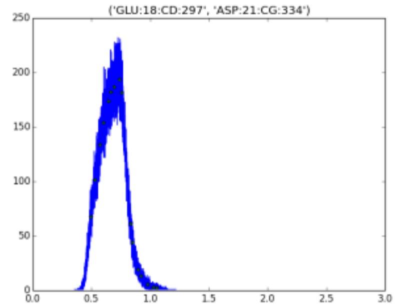

12. E18 CD – D21 CG

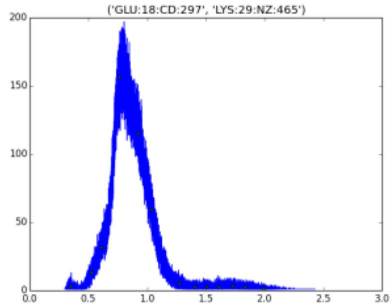

13. E18 CD – K29 NZ

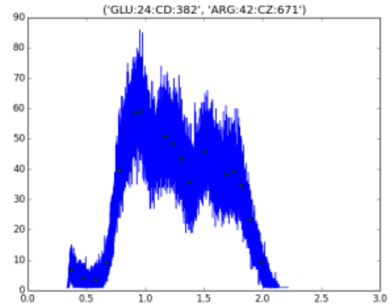

14. E24 CD – R42 CZ

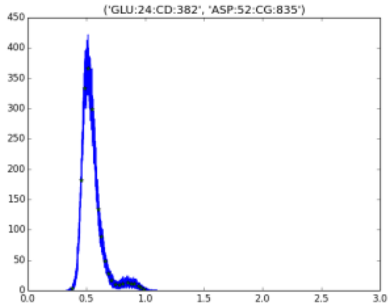

15. E24 CD – D52 CG

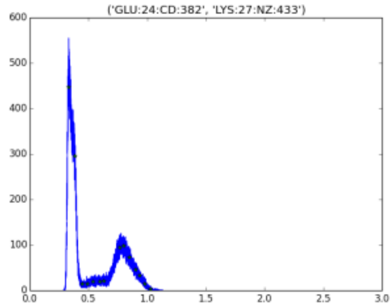

16. E24 CD – K27 NZ

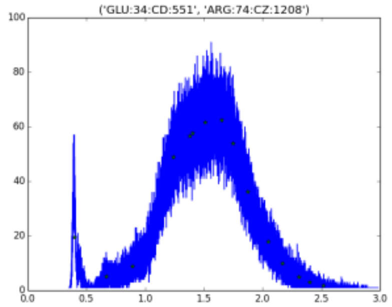

17. E34 CD – R74 CZ

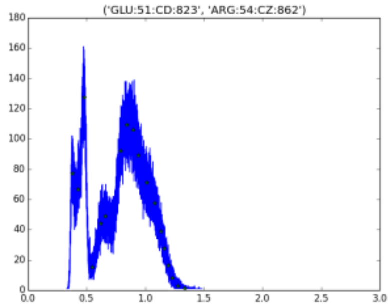

18. E51 CD – R54 CZ

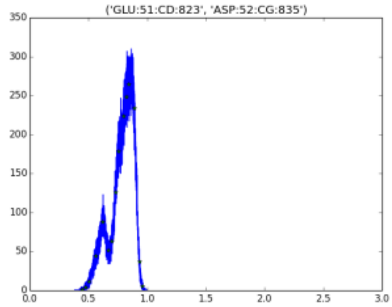

19. E51 CD – D52 CG

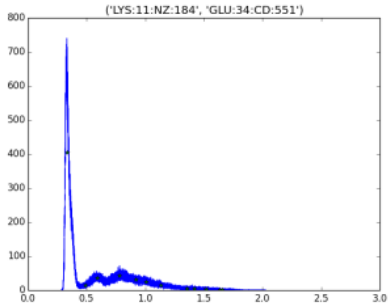

20. K11 NZ – E34 CD

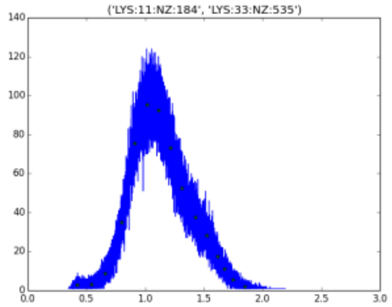

21. K11 NZ – K33 NZ

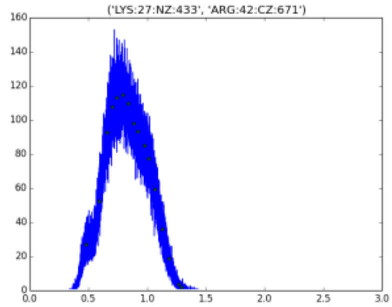

22. K27 NZ – R42 CZ

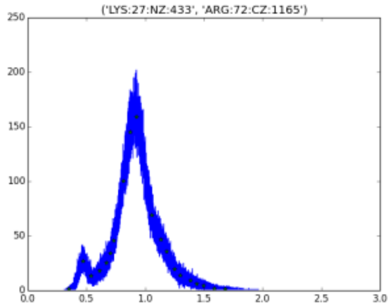

23. K27 NZ – R72 CZ

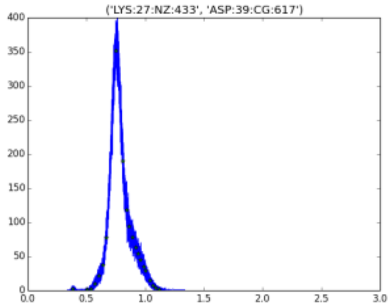

24. K27 NZ – D39 CG

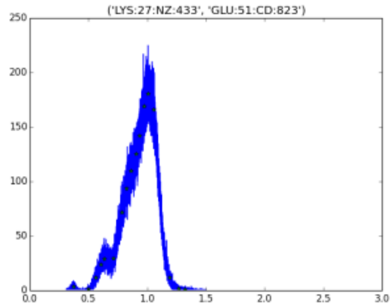

25. K27 NZ – E51 CD

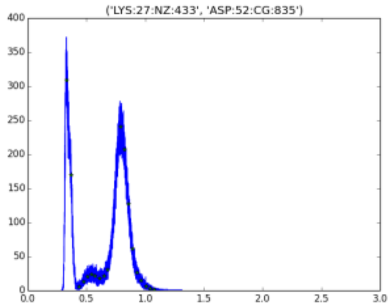

26. K27 NZ – D52 CG

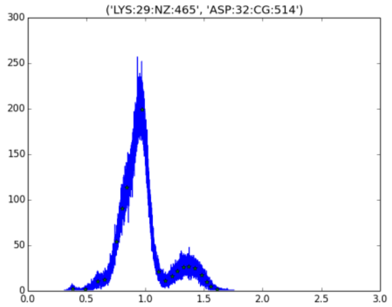

27. K29 NZ – D32 CG

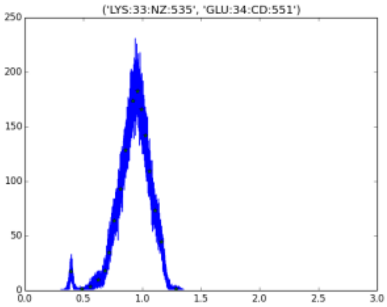

28. K33 NZ – E34 CD

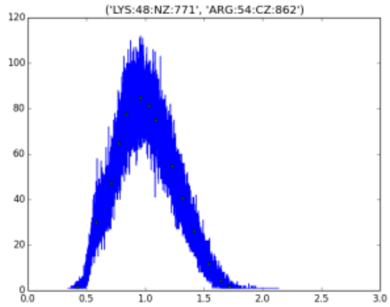

29. K48 NZ - R54 CZ

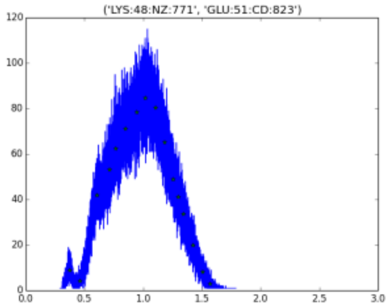

30. K48 NZ – E51 CD

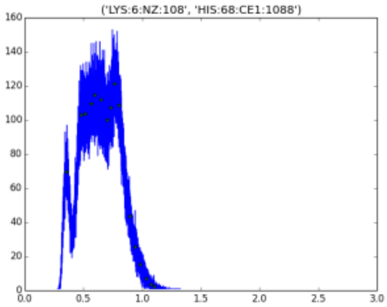

31. K6 NZ – H8 CE1

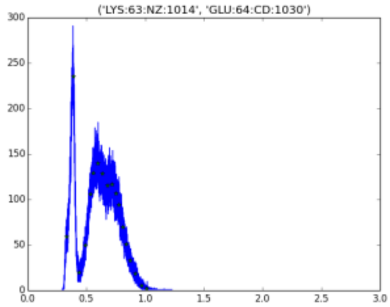

32. K63 NZ – E64 CD

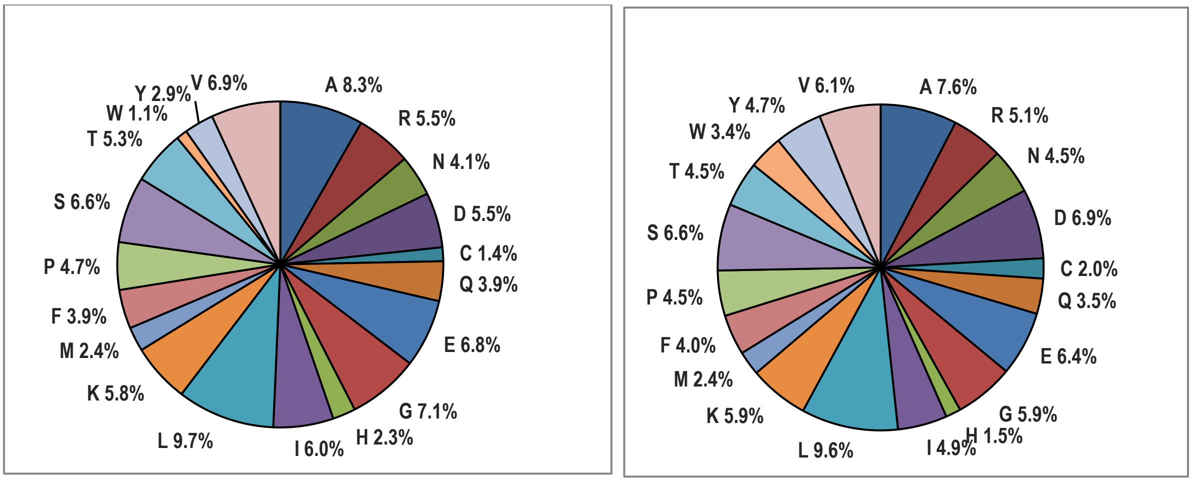

Figure S3: *left panel: average amino acid composition of proteins contained in release 2024_04 of UniProtKB, published on Jul 24, 2024 (https://www.uniprot.org/uniprotkb/statistics); right panel: amino acid composition of Ostrinia furnacalis chitinolytic enzyme (UniProtKB/Swiss-Prot ID: Q06GJ0)*

Figure S4: Binned distributions of close distance interactions between D, E, H, K, and R side chains in ofCE as calculated along the 30 ns MD trajectory with the g_distmap tool. Only side chain-side chain approaches exhibiting more than 5 % occurrences at r_{id} < 0.6 nm have been considered.

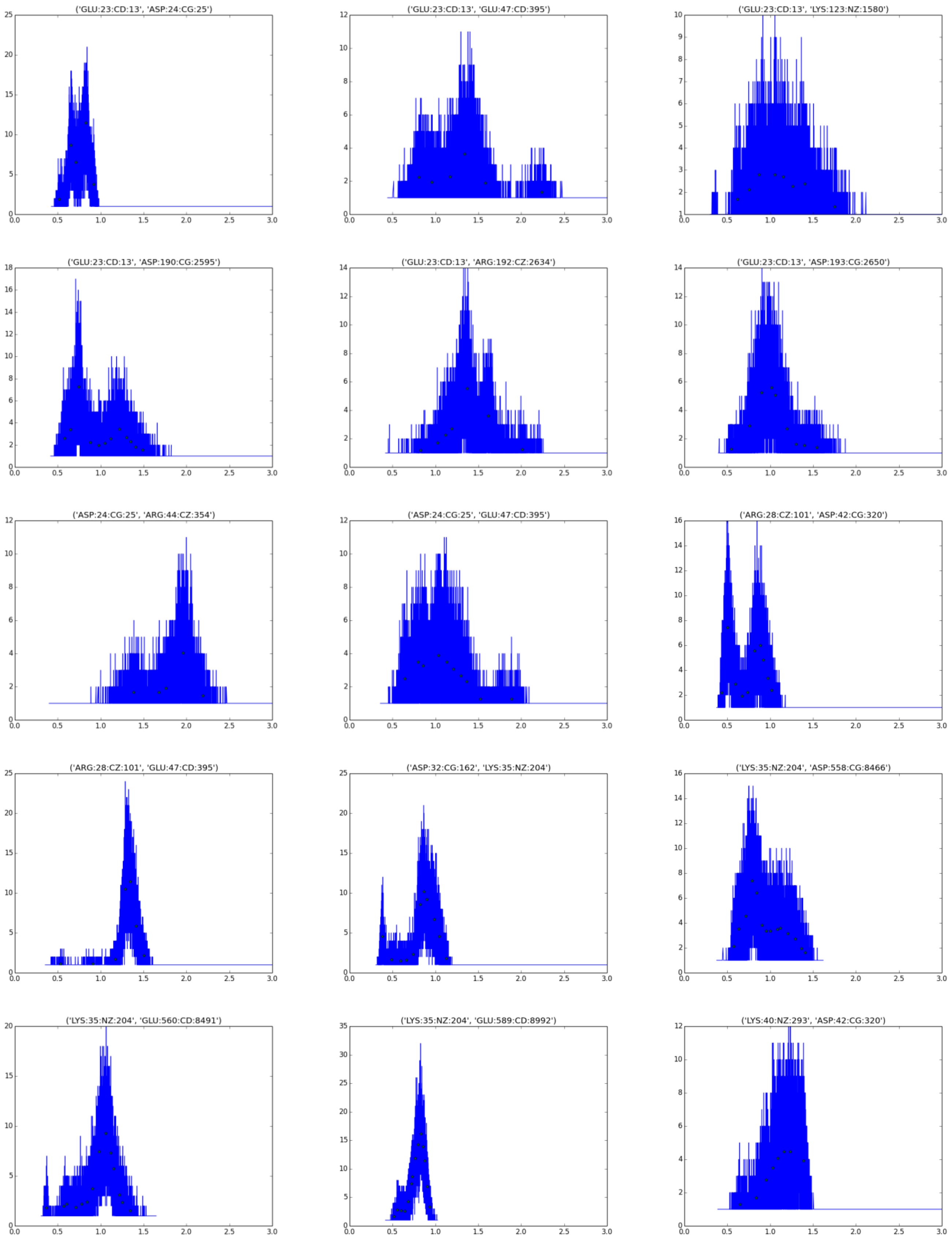

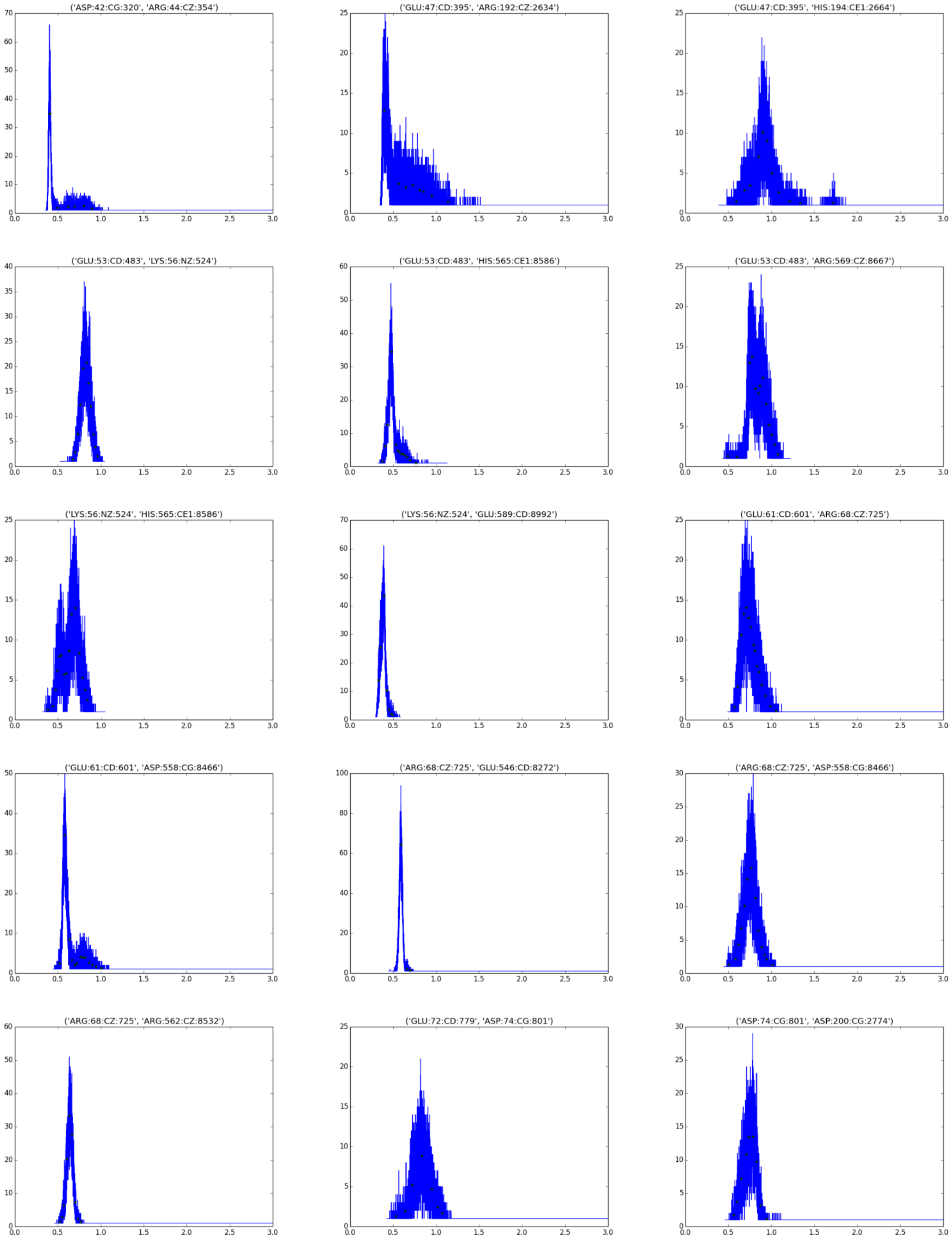
('ASP:42:CG:320', 'ARG:44:CZ:354')
('GLU:47:CD:395', 'ARG:192:CZ:2634')
('GLU:47:CD:395', 'HIS:194:CE1:2664')
('GLU:53:CD:483', 'LYS:56:NZ:524')
('GLU:53:CD:483', 'HIS:565:CE1:8586')
('GLU:53:CD:483', 'ARG:569:CZ:8667')
('LYS:56:NZ:524', 'HIS:565:CE1:8586')
('LYS:56:NZ:524', 'GLU:589:CD:8992')
('GLU:61:CD:601', 'ARG:68:CZ:725')
('GLU:61:CD:601', 'ASP:558:CG:8466')
('ARG:68:CZ:725', 'GLU:546:CD:8272')
('ARG:68:CZ:725', 'ASP:558:CG:8466')
('ARG:68:CZ:725', 'ARG:562:CZ:8532')
('GLU:72:CD:779', 'ASP:74:CG:801')
('ASP:74:CG:801', 'ASP:200:CG:2774')

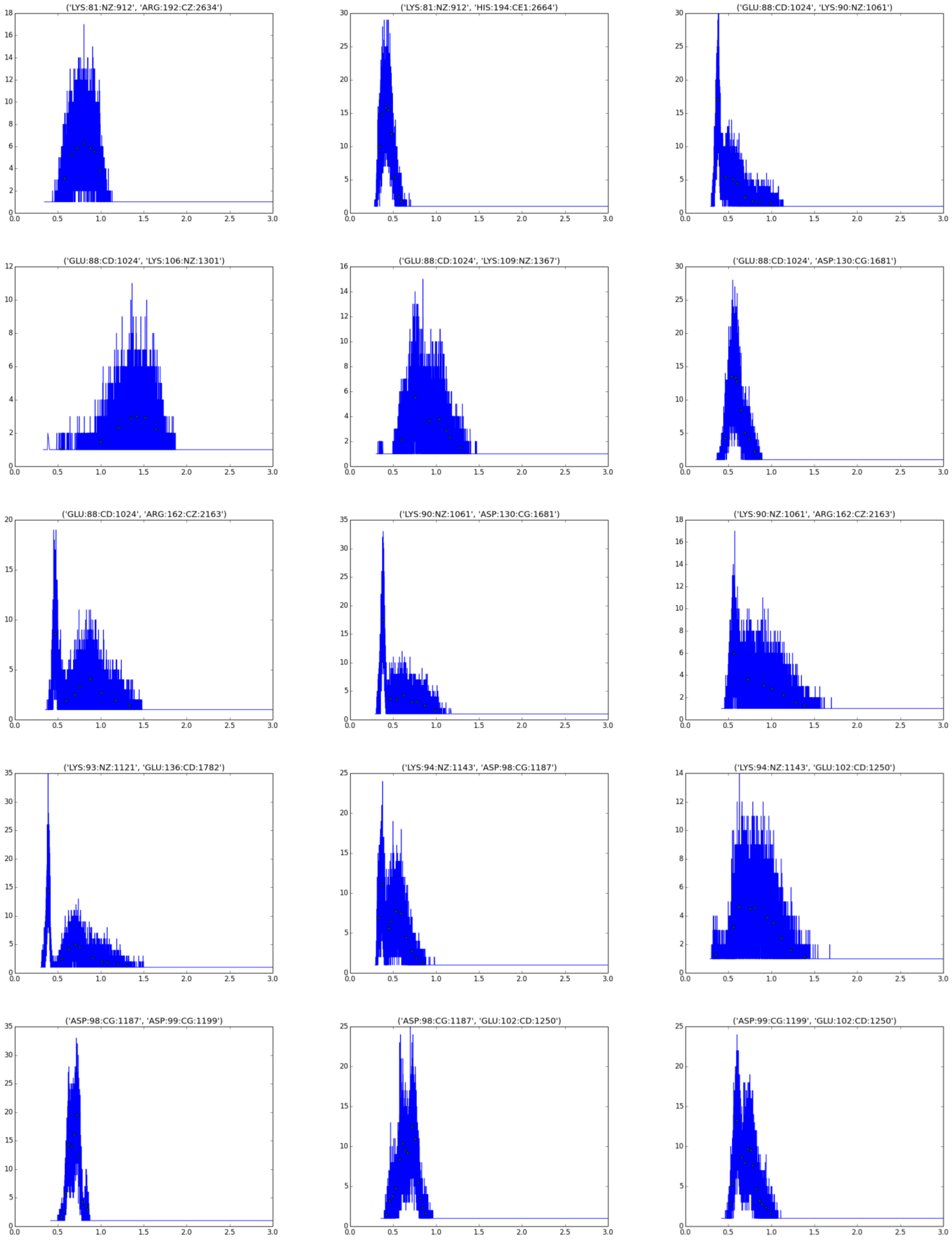
('LYS:81:NZ:912', 'ARG:192:CZ:2634')
('LYS:81:NZ:912', 'HIS:194:CE1:2664')
('GLU:88:CD:1024', 'LYS:90:NZ:1061')
('GLU:88:CD:1024', 'LYS:106:NZ:1301')
('GLU:88:CD:1024', 'LYS:109:NZ:1367')
('GLU:88:CD:1024', 'ASP:130:CG:1681')
('GLU:88:CD:1024', 'ARG:162:CZ:2163')
('LYS:90:NZ:1061', 'ASP:130:CG:1681')
('LYS:90:NZ:1061', 'ARG:162:CZ:2163')
('LYS:93:NZ:1121', 'GLU:136:CD:1782')
('LYS:94:NZ:1143', 'ASP:98:CG:1187')
('LYS:94:NZ:1143', 'GLU:102:CD:1250')
('ASP:98:CG:1187', 'ASP:99:CG:1199')
('ASP:98:CG:1187', 'GLU:102:CD:1250')
('ASP:99:CG:1199', 'GLU:102:CD:1250')

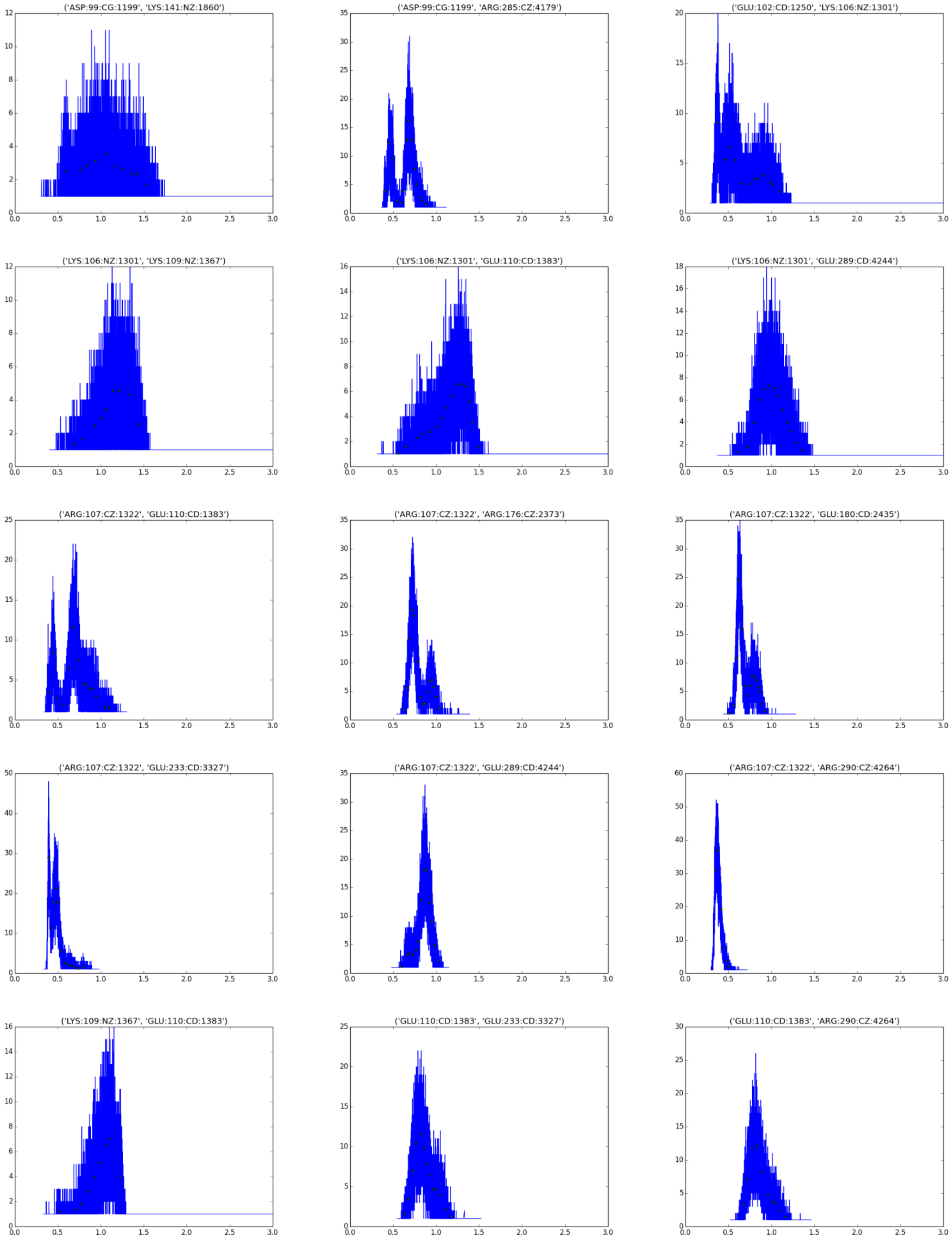
('ASP:99:CG:1199', 'LYS:141:NZ:1860')
('ASP:99:CG:1199', 'ARG:285:CZ:4179')
('GLU:102:CD:1250', 'LYS:106:NZ:1301')
('LYS:106:NZ:1301', 'LYS:109:NZ:1367')
('LYS:106:NZ:1301', 'GLU:110:CD:1383')
('LYS:106:NZ:1301', 'GLU:289:CD:4244')
('ARG:107:CZ:1322', 'GLU:110:CD:1383')
('ARG:107:CZ:1322', 'ARG:176:CZ:2373')
('ARG:107:CZ:1322', 'GLU:180:CD:2435')
('ARG:107:CZ:1322', 'GLU:233:CD:3327')
('ARG:107:CZ:1322', 'GLU:289:CD:4244')
('ARG:107:CZ:1322', 'ARG:290:CZ:4264')
('LYS:109:NZ:1367', 'GLU:110:CD:1383')
('GLU:110:CD:1383', 'GLU:233:CD:3327')
('GLU:110:CD:1383', 'ARG:290:CZ:4264')

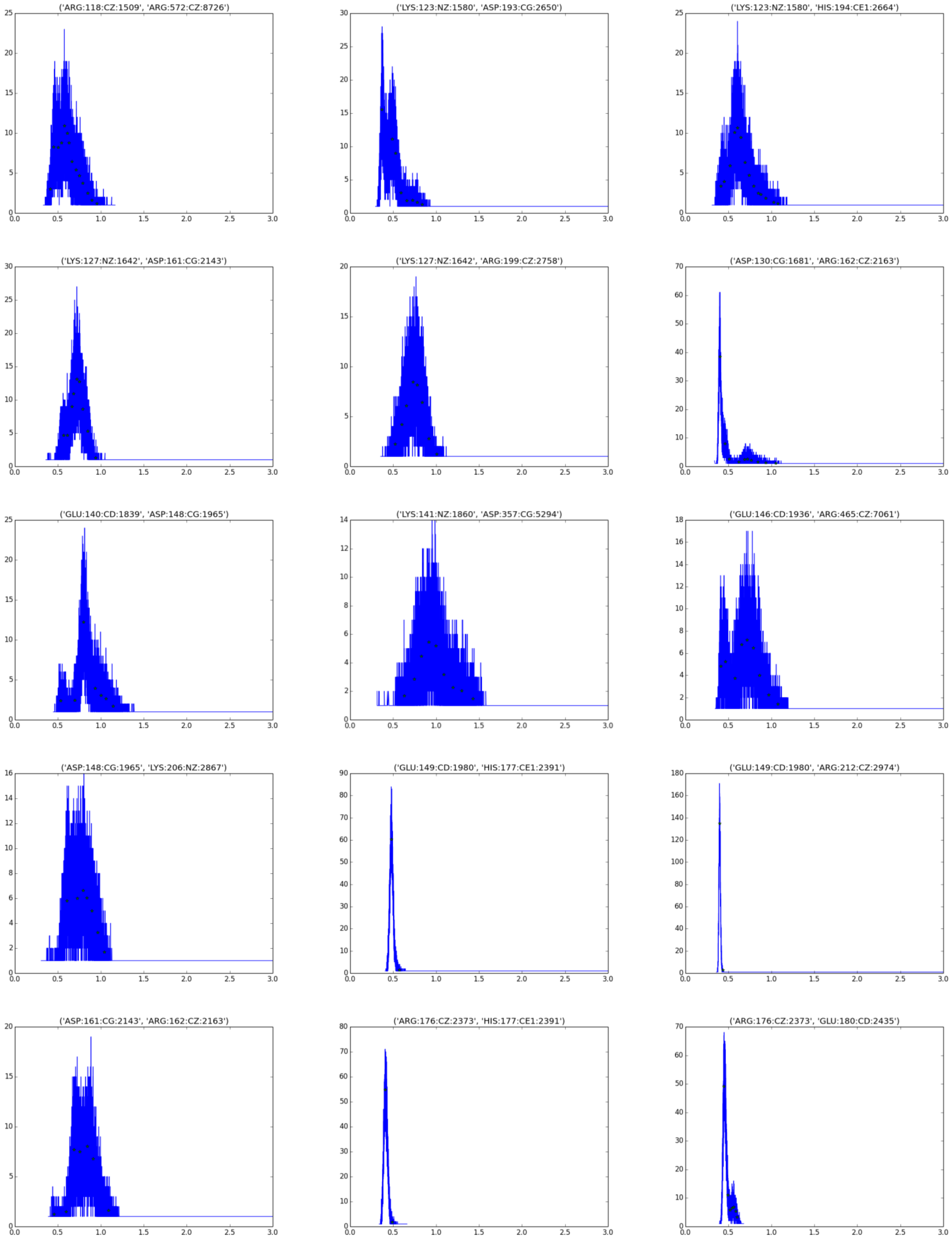
('ARG:118:CZ:1509', 'ARG:572:CZ:8726')
('LYS:123:NZ:1580', 'ASP:193:CG:2650')
('LYS:123:NZ:1580', 'HIS:194:CE1:2664')
('LYS:127:NZ:1642', 'ASP:161:CG:2143')
('LYS:127:NZ:1642', 'ARG:199:CZ:2758')
('ASP:130:CG:1681', 'ARG:162:CZ:2163')
('GLU:140:CD:1839', 'ASP:148:CG:1965')
('LYS:141:NZ:1860', 'ASP:357:CG:5294')
('GLU:146:CD:1936', 'ARG:465:CZ:7061')
('ASP:148:CG:1965', 'LYS:206:NZ:2867')
('GLU:149:CD:1980', 'HIS:177:CE1:2391')
('GLU:149:CD:1980', 'ARG:212:CZ:2974')
('ASP:161:CG:2143', 'ARG:162:CZ:2163')
('ARG:176:CZ:2373', 'HIS:177:CE1:2391')
('ARG:176:CZ:2373', 'GLU:180:CD:2435')
0.0
0.5
1.0
1.5
2.0
2.5
3.0

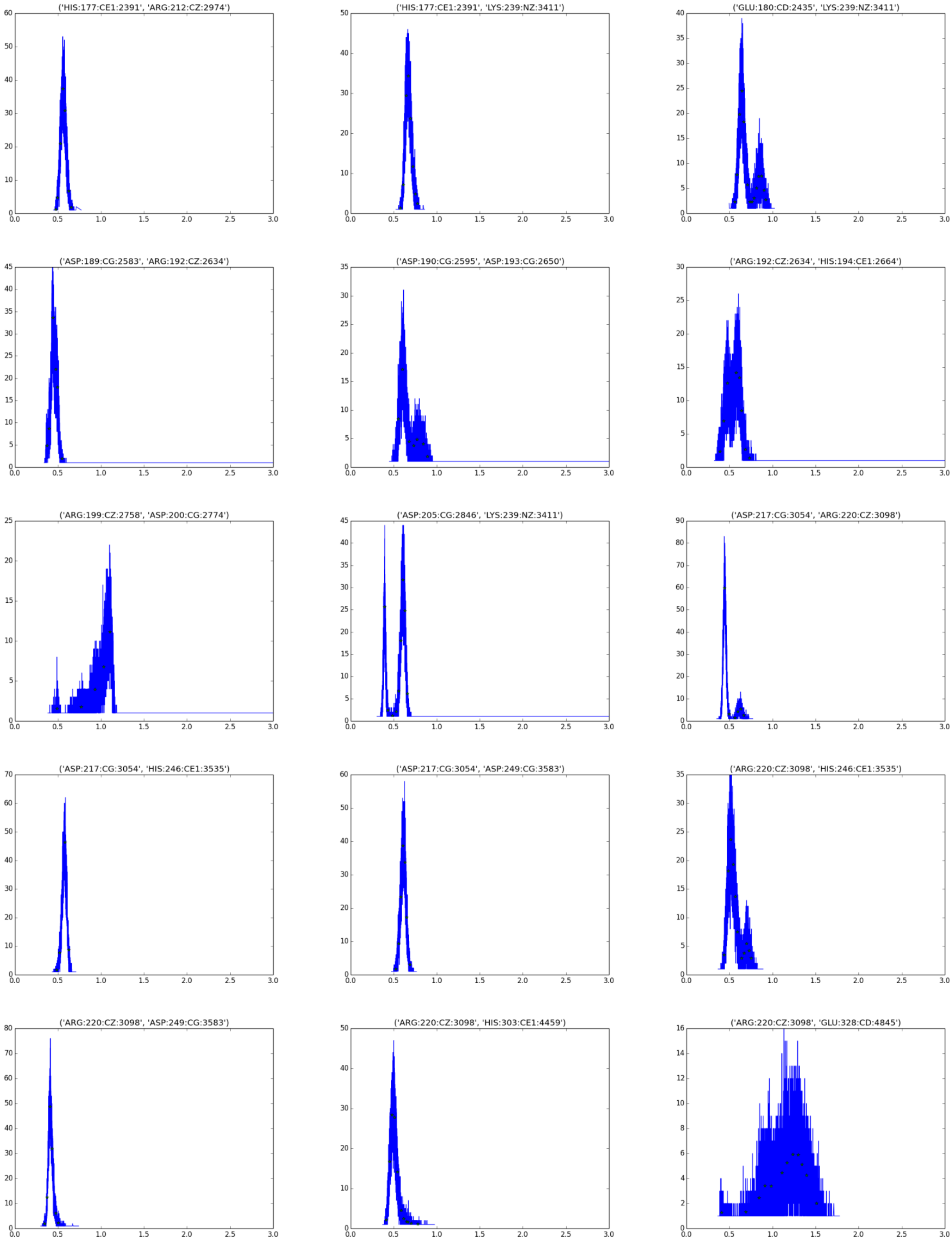
('HIS:177:CE1:2391', 'ARG:212:CZ:2974')
('HIS:177:CE1:2391', 'LYS:239:NZ:3411')
('GLU:180:CD:2435', 'LYS:239:NZ:3411')
('ASP:189:CG:2583', 'ARG:192:CZ:2634')
('ASP:190:CG:2595', 'ASP:193:CG:2650')
('ARG:192:CZ:2634', 'HIS:194:CE1:2664')
('ARG:199:CZ:2758', 'ASP:200:CG:2774')
('ASP:205:CG:2846', 'LYS:239:NZ:3411')
('ASP:217:CG:3054', 'ARG:220:CZ:3098')
('ASP:217:CG:3054', 'HIS:246:CE1:3535')
('ASP:217:CG:3054', 'ASP:249:CG:3583')
('ARG:220:CZ:3098', 'HIS:246:CE1:3535')
('ARG:220:CZ:3098', 'ASP:249:CG:3583')
('ARG:220:CZ:3098', 'HIS:303:CE1:4459')
('ARG:220:CZ:3098', 'GLU:328:CD:4845')

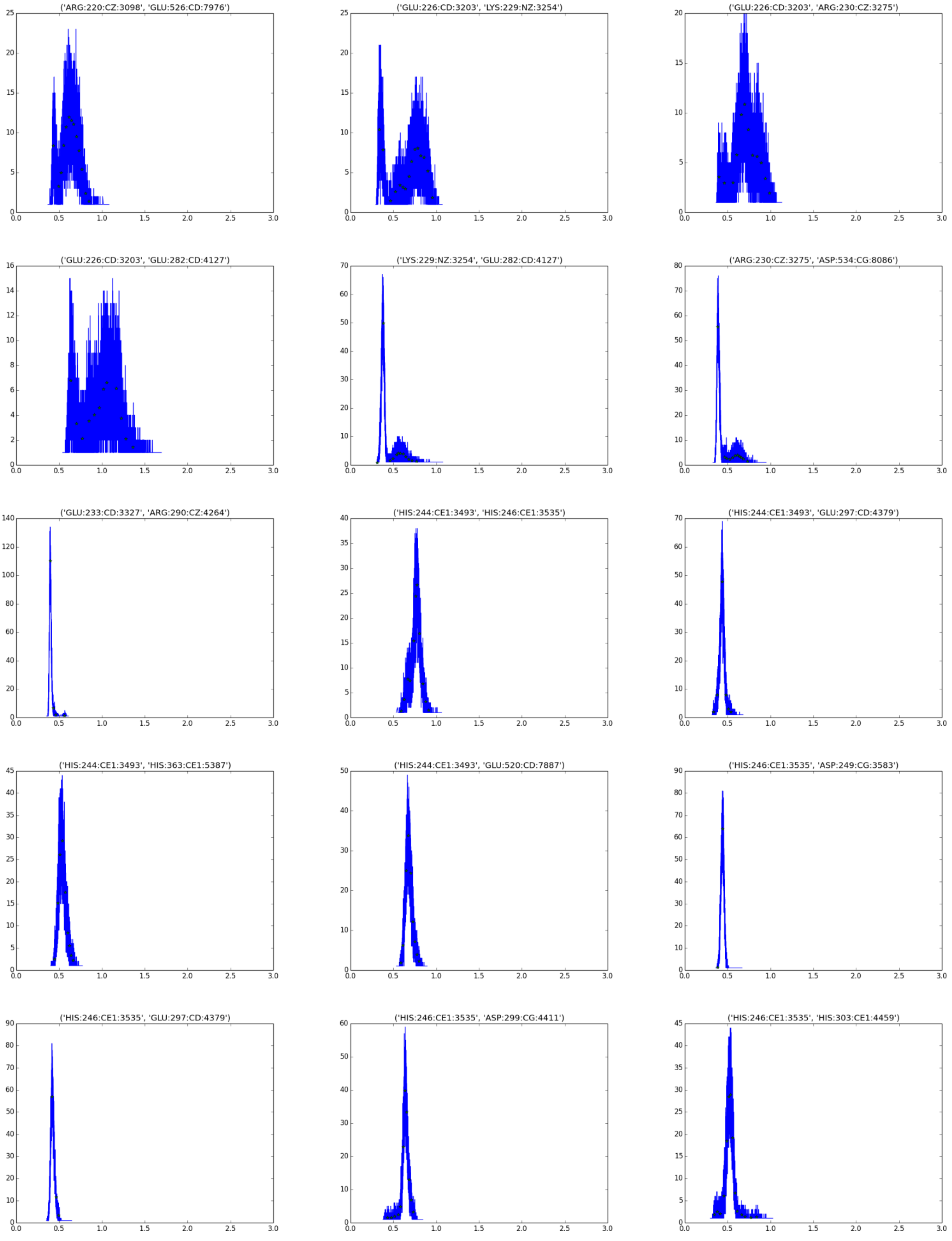
('ARG:220:CZ:3098', 'GLU:526:CD:7976')
('GLU:226:CD:3203', 'LYS:229:NZ:3254')
('GLU:226:CD:3203', 'ARG:230:CZ:3275')
('GLU:226:CD:3203', 'GLU:282:CD:4127')
('LYS:229:NZ:3254', 'GLU:282:CD:4127')
('ARG:230:CZ:3275', 'ASP:534:CG:8086')
('GLU:233:CD:3327', 'ARG:290:CZ:4264')
('HIS:244:CE1:3493', 'HIS:246:CE1:3535')
('HIS:244:CE1:3493', 'GLU:297:CD:4379')
('HIS:244:CE1:3493', 'HIS:363:CE1:5387')
('HIS:244:CE1:3493', 'GLU:520:CD:7887')
('HIS:246:CE1:3535', 'ASP:249:CG:3583')
('HIS:246:CE1:3535', 'GLU:297:CD:4379')
('HIS:246:CE1:3535', 'ASP:299:CG:4411')
('HIS:246:CE1:3535', 'HIS:303:CE1:4459')

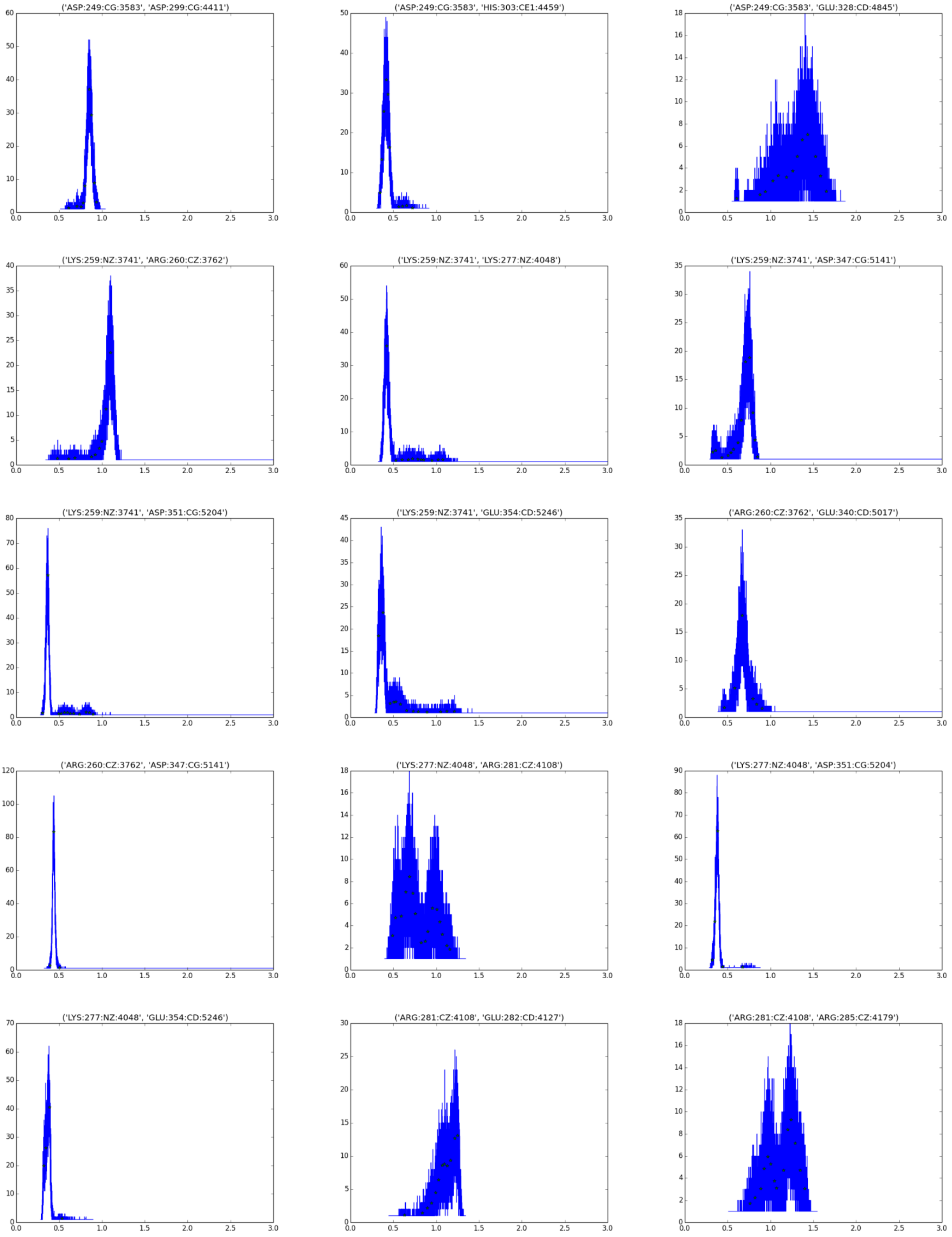
('ASP:249:CG:3583', 'ASP:299:CG:4411')
('ASP:249:CG:3583', 'HIS:303:CE1:4459')
('ASP:249:CG:3583', 'GLU:328:CD:4845')
('LYS:259:NZ:3741', 'ARG:260:CZ:3762')
('LYS:259:NZ:3741', 'LYS:277:NZ:4048')
('LYS:259:NZ:3741', 'ASP:347:CG:5141')
('LYS:259:NZ:3741', 'ASP:351:CG:5204')
('LYS:259:NZ:3741', 'GLU:354:CD:5246')
('ARG:260:CZ:3762', 'GLU:340:CD:5017')
('ARG:260:CZ:3762', 'ASP:347:CG:5141')
('LYS:277:NZ:4048', 'ARG:281:CZ:4108')
('LYS:277:NZ:4048', 'ASP:351:CG:5204')
('LYS:277:NZ:4048', 'GLU:354:CD:5246')
('ARG:281:CZ:4108', 'GLU:282:CD:4127')
('ARG:281:CZ:4108', 'ARG:285:CZ:4179')

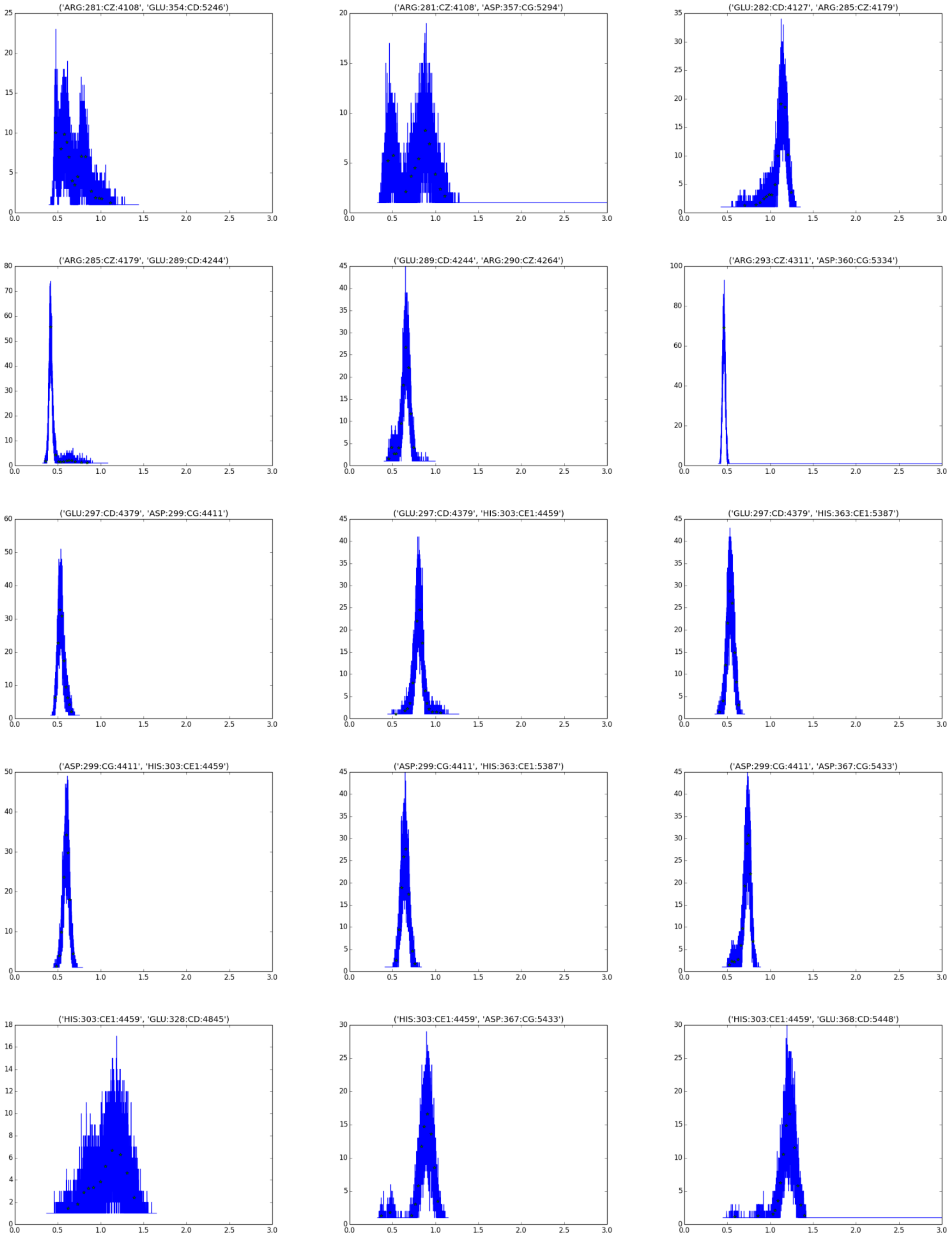
('ARG:281:CZ:4108', 'GLU:354:CD:5246')
('ARG:281:CZ:4108', 'ASP:357:CG:5294')
('GLU:282:CD:4127', 'ARG:285:CZ:4179')
('ARG:285:CZ:4179', 'GLU:289:CD:4244')
('GLU:289:CD:4244', 'ARG:290:CZ:4264')
('ARG:293:CZ:4311', 'ASP:360:CG:5334')
('GLU:297:CD:4379', 'ASP:299:CG:4411')
('GLU:297:CD:4379', 'HIS:303:CE1:4459')
('GLU:297:CD:4379', 'HIS:363:CE1:5387')
('ASP:299:CG:4411', 'HIS:303:CE1:4459')
('ASP:299:CG:4411', 'HIS:363:CE1:5387')
('ASP:299:CG:4411', 'ASP:367:CG:5433')
('HIS:303:CE1:4459', 'GLU:328:CD:4845')
('HIS:303:CE1:4459', 'ASP:367:CG:5433')
('HIS:303:CE1:4459', 'GLU:368:CD:5448')

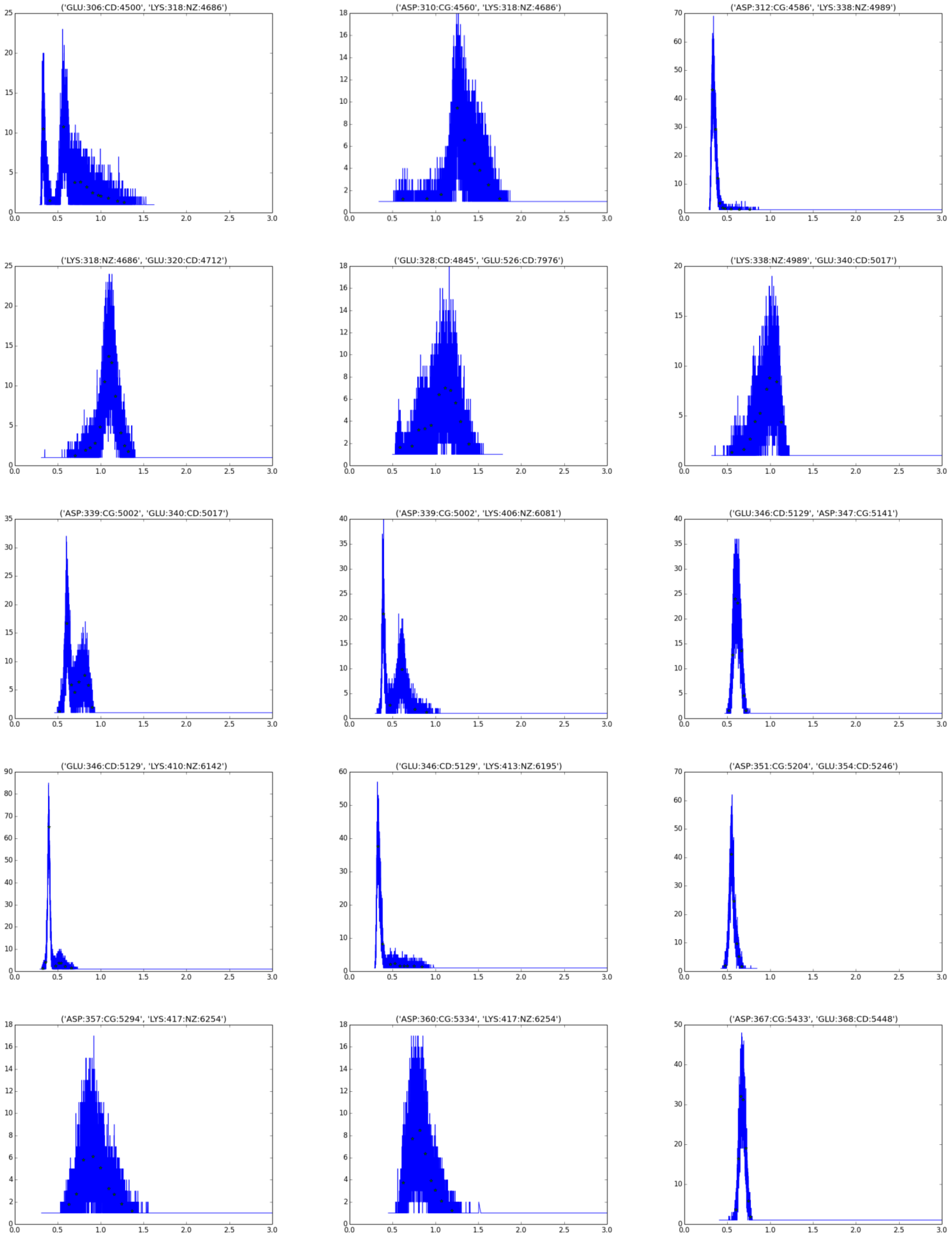
('GLU:306:CD:4500', 'LYS:318:NZ:4686')
('ASP:310:CG:4560', 'LYS:318:NZ:4686')
('ASP:312:CG:4586', 'LYS:338:NZ:4989')
('LYS:318:NZ:4686', 'GLU:320:CD:4712')
('GLU:328:CD:4845', 'GLU:526:CD:7976')
('LYS:338:NZ:4989', 'GLU:340:CD:5017')
('ASP:339:CG:5002', 'GLU:340:CD:5017')
('ASP:339:CG:5002', 'LYS:406:NZ:6081')
('GLU:346:CD:5129', 'ASP:347:CG:5141')
('GLU:346:CD:5129', 'LYS:410:NZ:6142')
('GLU:346:CD:5129', 'LYS:413:NZ:6195')
('ASP:351:CG:5204', 'GLU:354:CD:5246')
('ASP:357:CG:5294', 'LYS:417:NZ:6254')
('ASP:360:CG:5334', 'LYS:417:NZ:6254')
('ASP:367:CG:5433', 'GLU:368:CD:5448')

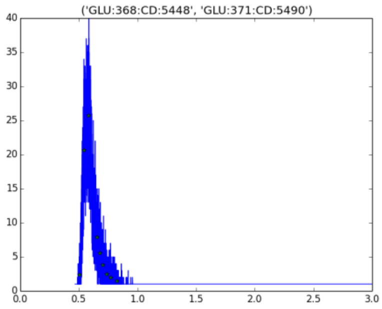
('GLU:368:CD:5448', 'GLU:371:CD:5490')
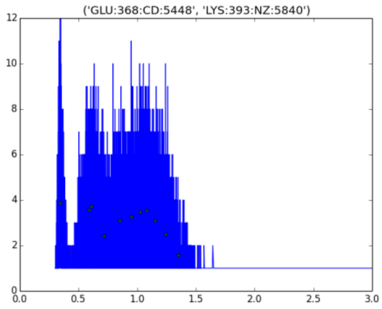
('GLU:368:CD:5448', 'LYS:393:NZ:5840')
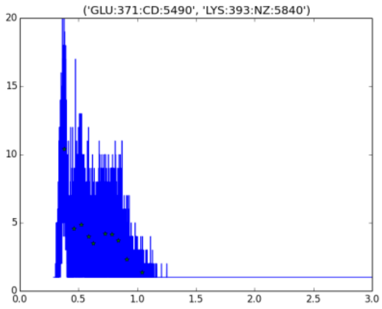
('GLU:371:CD:5490', 'LYS:393:NZ:5840')
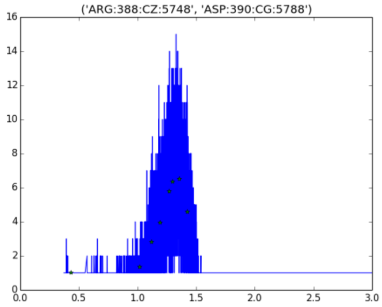
('ARG:388:CZ:5748', 'ASP:390:CG:5788')
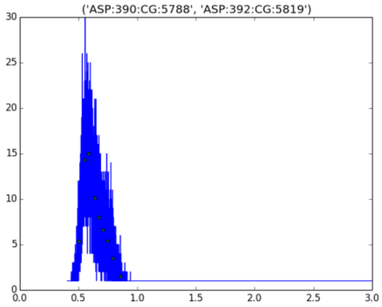
('ASP:390:CG:5788', 'ASP:392:CG:5819')
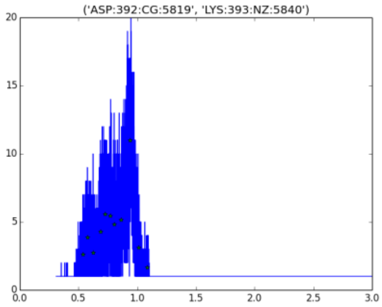
('ASP:392:CG:5819', 'LYS:393:NZ:5840')
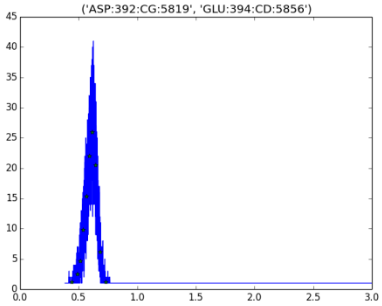
('ASP:392:CG:5819', 'GLU:394:CD:5856')
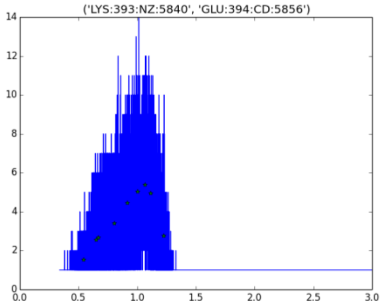
('LYS:393:NZ:5840', 'GLU:394:CD:5856')
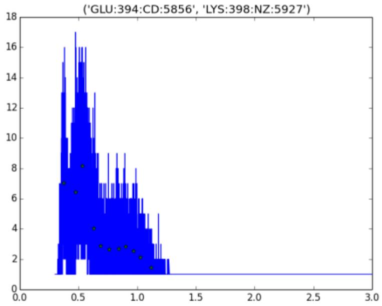
('GLU:394:CD:5856', 'LYS:398:NZ:5927')
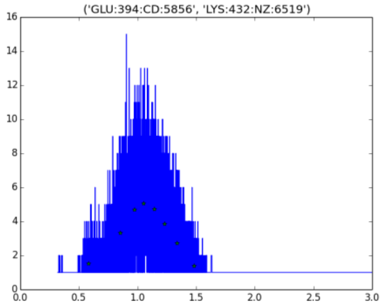
('GLU:394:CD:5856', 'LYS:432:NZ:6519')
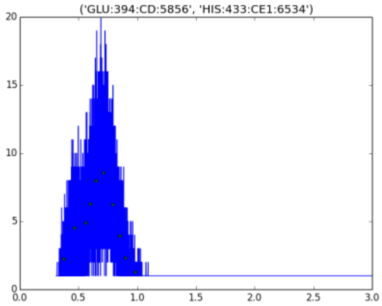
('GLU:394:CD:5856', 'HIS:433:CE1:6534')
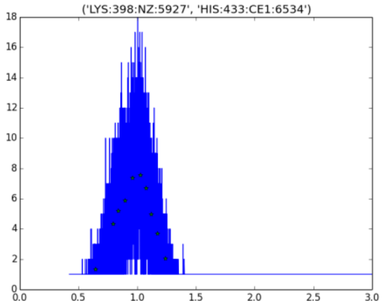
('LYS:398:NZ:5927', 'HIS:433:CE1:6534')
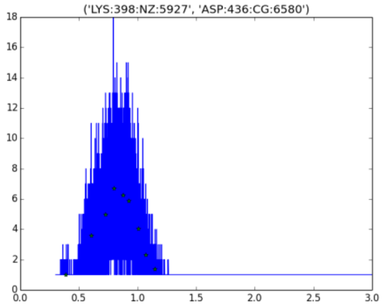
('LYS:398:NZ:5927', 'ASP:436:CG:6580')
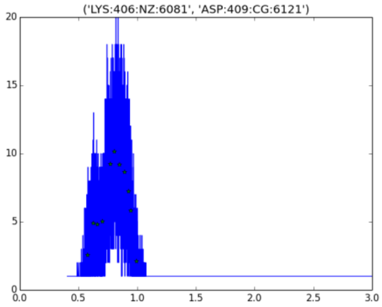
('LYS:406:NZ:6081', 'ASP:409:CG:6121')
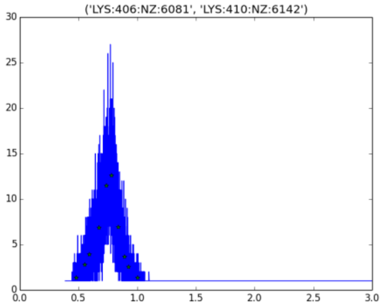
('LYS:406:NZ:6081', 'LYS:410:NZ:6142')

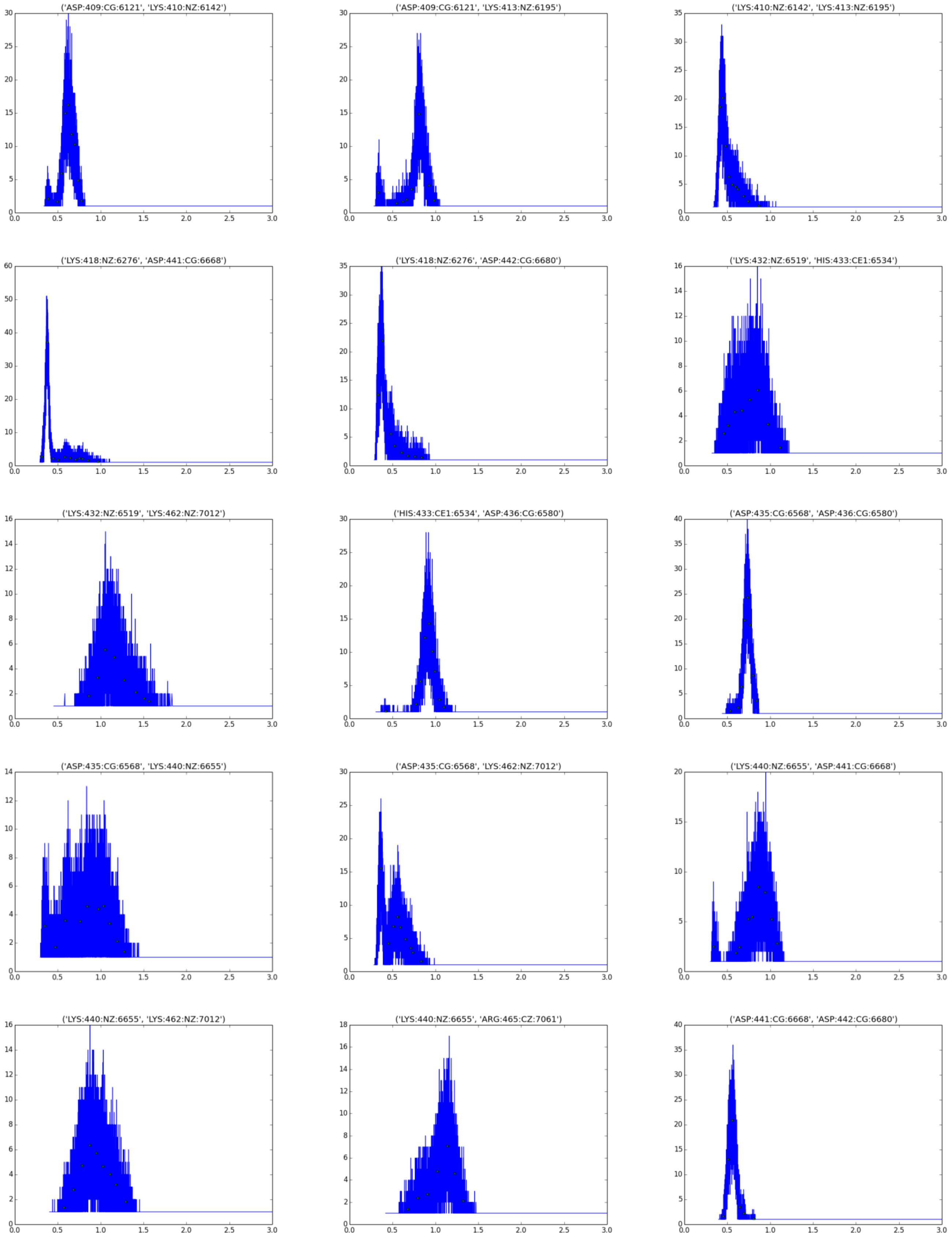
('ASP:409:CG:6121', 'LYS:410:NZ:6142')
('ASP:409:CG:6121', 'LYS:413:NZ:6195')
('LYS:410:NZ:6142', 'LYS:413:NZ:6195')
('LYS:418:NZ:6276', 'ASP:441:CG:6668')
('LYS:418:NZ:6276', 'ASP:442:CG:6680')
('LYS:432:NZ:6519', 'HIS:433:CE1:6534')
('LYS:432:NZ:6519', 'LYS:462:NZ:7012')
('HIS:433:CE1:6534', 'ASP:436:CG:6580')
('ASP:435:CG:6568', 'ASP:436:CG:6580')
('ASP:435:CG:6568', 'LYS:440:NZ:6655')
('ASP:435:CG:6568', 'LYS:462:NZ:7012')
('LYS:440:NZ:6655', 'ASP:441:CG:6668')
('LYS:440:NZ:6655', 'LYS:462:NZ:7012')
('LYS:440:NZ:6655', 'ARG:465:CZ:7061')
('ASP:441:CG:6668', 'ASP:442:CG:6680')

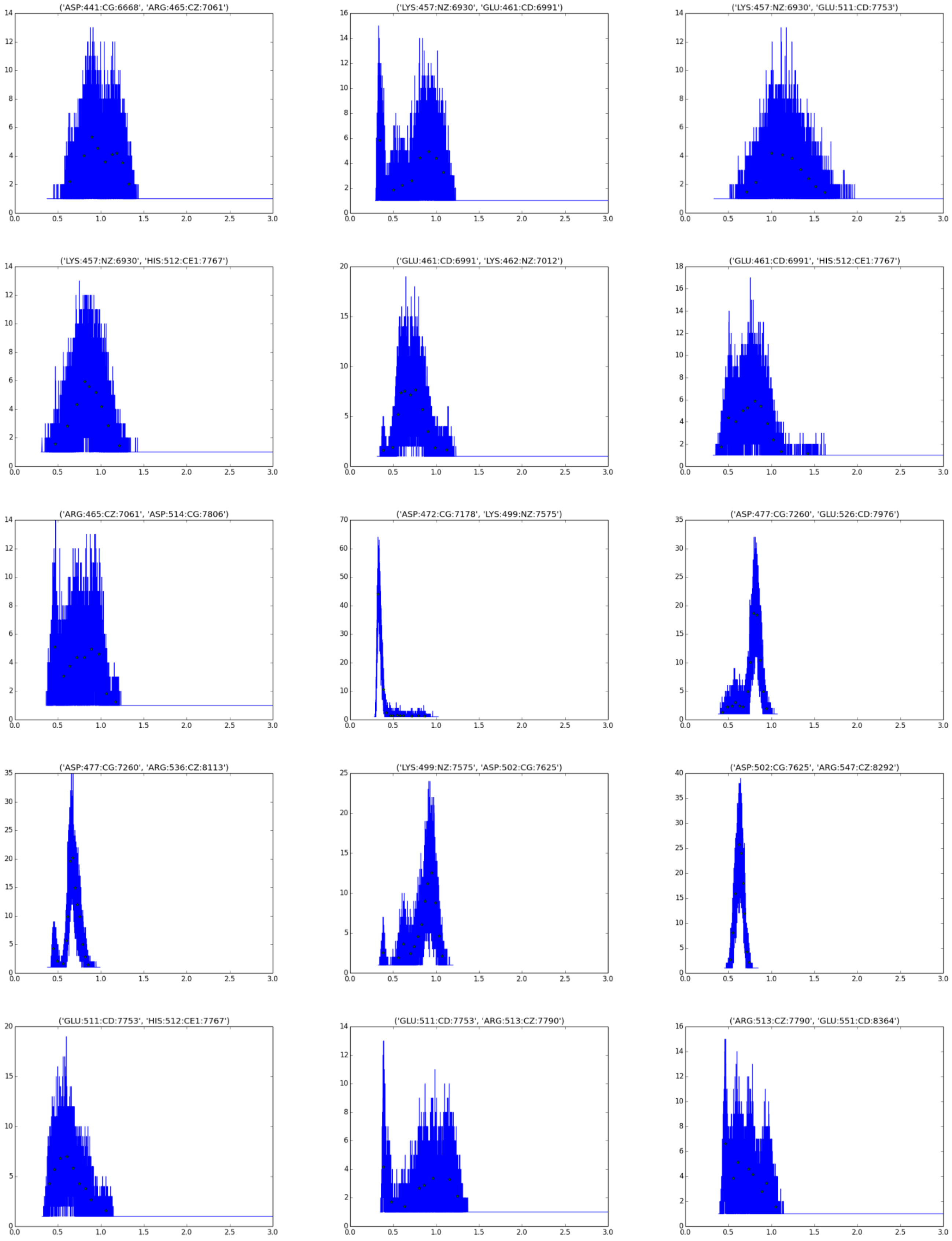
('ASP:441:CG:6668', 'ARG:465:CZ:7061')
('LYS:457:NZ:6930', 'GLU:461:CD:6991')
('LYS:457:NZ:6930', 'GLU:511:CD:7753')
('LYS:457:NZ:6930', 'HIS:512:CE1:7767')
('GLU:461:CD:6991', 'LYS:462:NZ:7012')
('GLU:461:CD:6991', 'HIS:512:CE1:7767')
('ARG:465:CZ:7061', 'ASP:514:CG:7806')
('ASP:472:CG:7178', 'LYS:499:NZ:7575')
('ASP:477:CG:7260', 'GLU:526:CD:7976')
('ASP:477:CG:7260', 'ARG:536:CZ:8113')
('LYS:499:NZ:7575', 'ASP:502:CG:7625')
('ASP:502:CG:7625', 'ARG:547:CZ:8292')
('GLU:511:CD:7753', 'HIS:512:CE1:7767')
('GLU:511:CD:7753', 'ARG:513:CZ:7790')
('ARG:513:CZ:7790', 'GLU:551:CD:8364')

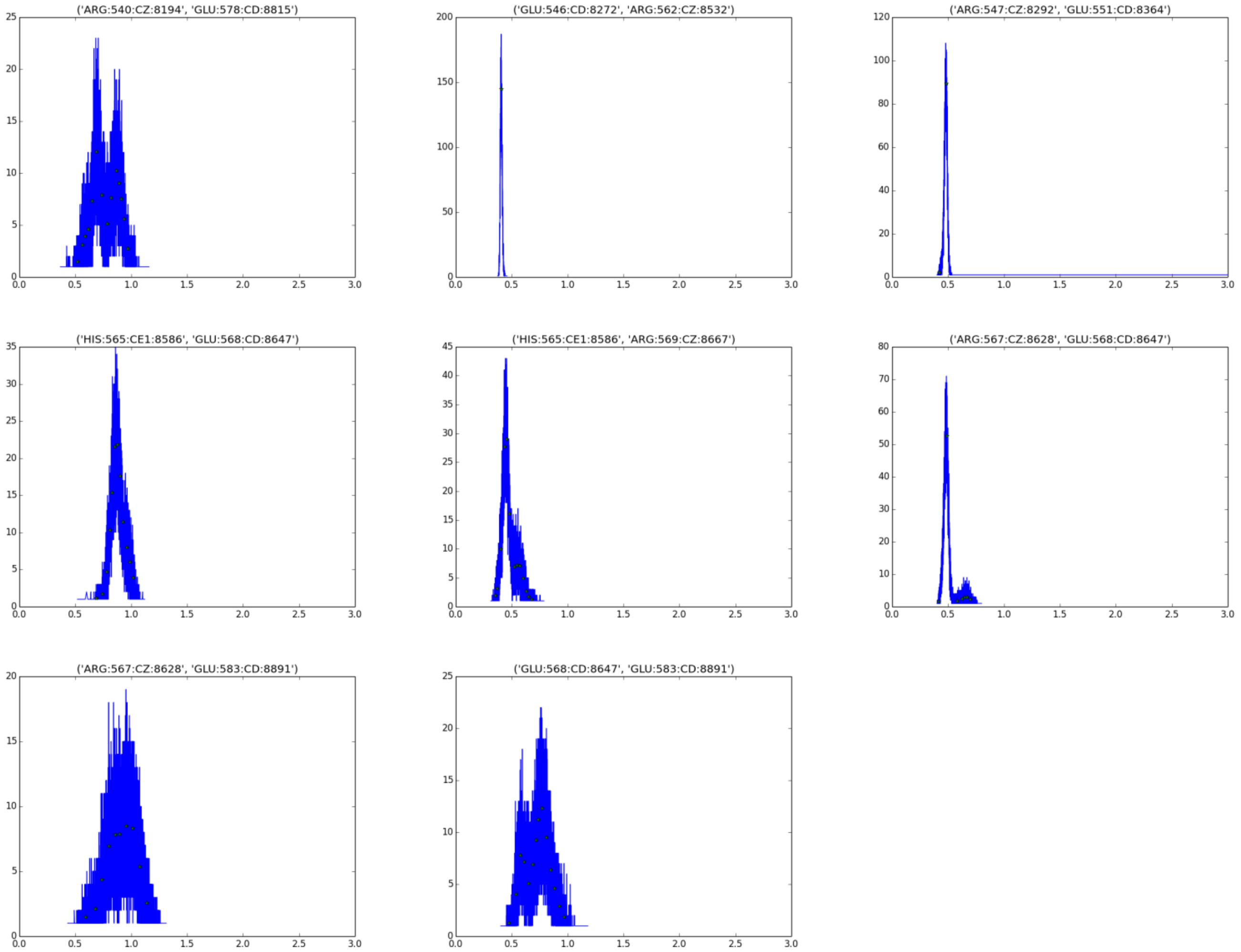
('ARG:540:CZ:8194', 'GLU:578:CD:8815')
0 5 10 15 20 25
0.0 0.5 1.0 1.5 2.0 2.5 3.0
('GLU:546:CD:8272', 'ARG:562:CZ:8532')
0 50 100 150 200
0.0 0.5 1.0 1.5 2.0 2.5 3.0
('ARG:547:CZ:8292', 'GLU:551:CD:8364')
0 20 40 60 80 100 120
0.0 0.5 1.0 1.5 2.0 2.5 3.0
('HIS:565:CE1:8586', 'GLU:568:CD:8647')
0 5 10 15 20 25 30 35
0.0 0.5 1.0 1.5 2.0 2.5 3.0
('HIS:565:CE1:8586', 'ARG:569:CZ:8667')
0 5 10 15 20 25 30 35 40 45
0.0 0.5 1.0 1.5 2.0 2.5 3.0
('ARG:567:CZ:8628', 'GLU:568:CD:8647')
0 10 20 30 40 50 60 70 80
0.0 0.5 1.0 1.5 2.0 2.5 3.0
('ARG:567:CZ:8628', 'GLU:583:CD:8891')
0 5 10 15 20
0.0 0.5 1.0 1.5 2.0 2.5 3.0
('GLU:568:CD:8647', 'GLU:583:CD:8891')
0 5 10 15 20 25
0.0 0.5 1.0 1.5 2.0 2.5 3.0

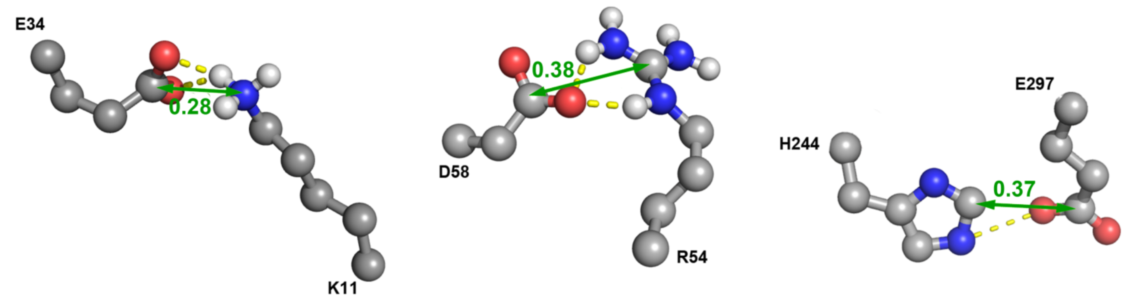

Figure S5: *Closest approaches of charged side chains stabilized by the formation of hydrogen bonding. Green arrows highlight the distances* r_{id} *of E34 CG, D58 CD, and E297 CG atoms respectively with K11 NZ, R54 CZ, and H244 CE1 in Ostrinia furnacalis chitinolytic enzyme.*

Table S1

Atom	# edges	$D_{i,12}$[a]	DSSP[b]
CD E23	6	1.55	N/A
CG D24	3	1.33	N/A
CZ R28	2	0.96	E
CG D32	1	1.30	E
NZ K35	4	1.17	E
NZ K38	0	1.00	E
NZ K40	1	1.42	E
CG D42	3	1.30	N/A
CZ R44	2	1.77	S
CD E47	5	1.17	S
CD E53	3	0.19	H
NZ K56	3	0.21	H
CD E61	2	0.93	T
CZ R68	4	0.66	N/A
CD E72	1	1.24	N/A
CG D74	2	1.03	E
NZ K81	2	1.10	E
CD E88	5	1.10	E
NZ K90	3	1.16	E
NZ K93	1	1.25	S
NZ K94	2	1.43	N/A
CG D98	3	1.10	H
CG D99	4	1.00	H
CD E102	4	1.10	H
NZ K106	5	1.28	H
CZ R107	6	0.43	T
NZ K109	3	1.00	H
CD E110	5	0.90	H
CZ R118	1	1.10	T
NZ K123	3	1.17	N/A
NZ K127	2	0.99	E
CG D130	3	0.87	E
CD E136	1	1.11	S
CD E140	1	1.01	N/A
NZ K141	2	0.98	N/A
CD E146	1	1.03	T
CG D148	2	0.83	N/A
CD E149	2	0.22	N/A
CZ R154	0	1.06	E
CG D161	2	1.38	T
CZ R162	4	1.11	E
CZ R176	3	0.05	H
CE1 H177	4	0.05	H
CD E180	3	0.07	H
CG D189	1	0.54	E
CG D190	2	0.51	T
CZ R192	5	0.78	T
CG D193	3	0.96	T
CE1 H194	4	0.95	E
CZ R199	2	1.13	S
CG D200	2	0.96	S
CG D205	1	0.37	E
NZ K206	1	1.19	N/A
CZ R212	2	0.13	E
CG D217	3	0.14	E
CZ R220	6	0.23	S
CD E226	3	1.15	H
NZ K229	2	0.94	H
CZ R230	2	0.74	H
CD E233	3	0.51	H
NZ K239	3	0.03	T
CE1 H244	4	0.02	E
CE1 H246	7	0.06	E
CG D249	6	0.14	N/A
NZ K259	5	1.05	S
CZ R260	3	0.89	S
NZ K265	0	1.20	H
NZ K273	0	1.15	S
NZ K277	4	1.18	H
CZ R281	5	1.10	H
CD E282	4	1.08	H
CZ R285	4	0.87	H
CD E289	4	0.68	H
CZ R290	4	0.49	T
CZ R293	1	0.22	E
CD E297	5	0.04	E
CG D299	6	0.06	E
CE1 H303	8	0.22	S
CD E306	1	0.77	T
CG D310	1	1.30	T
CG D312	1	1.11	N/A
NZ K318	3	1.24	T
CD E320	1	1.30	S
NZ K323	0	1.34	G
CD E328	4	0.43	S
NZ K338	2	1.01	N/A
CG D339	2	1.09	T
CD E340	3	1.05	H
CD E346	3	0.95	H
CG D347	3	0.80	H
CG D351	3	0.65	H
CD E354	4	1.04	H
CG D357	3	0.77	N/A
CG D360	2	0.45	S
CE1 H363	3	0.04	E
CG D367	3	0.18	N/A
CD E368	4	0.56	N/A
CD E371	2	0.83	H
CG D378	0	1.25	H
CZ R388	1	1.57	T
CG D390	2	1.29	N/A
CG D392	3	1.21	S
NZ K393	4	1.19	S
CD E394	5	1.18	S
NZ K398	3	1.05	H
NZ K406	3	0.93	H
CG D409	3	0.97	H
NZ K410	4	0.98	H
NZ K413	3	1.36	H
NZ K417	2	0.94	S
NZ K418	2	1.12	N/A
NZ K432	3	1.27	T
CE1 H433	4	0.81	T
CG D435	3	0.97	H
CG D436	3	1.08	H
NZ K440	4	1.21	T
CG D441	4	1.15	T
CG D442	2	0.88	T
CG D453	0	0.91	N/A
NZ K457	3	1.29	H
CD E461	3	1.16	H
NZ K462	4	1.16	T
CZ R465	4	0.64	E
CG D472	1	0.74	H
CG D477	2	0.26	T
NZ K499	2	0.91	H
CG D502	2	0.80	H
CD E511	3	1.31	G
CE1 H512	3	1.10	G
CZ R513	2	1.22	G
CG D514	1	1.02	G
CD E520	1	0.01	E
CD E526	3	0.36	T
CG D529	0	0.88	N/A
CG D534	1	0.57	H
CZ R536	1	0.64	H
CZ R540	1	0.34	H
CD E546	2	0.18	H
CZ R547	2	0.52	H
CD E551	2	0.54	N/A
CG D558	3	0.95	G
CD E560	1	0.58	H
CZ R562	2	0.25	H
CE1 H565	4	0.10	H
CZ R567	2	0.75	H
CD E568	3	0.53	H
CZ R569	2	0.13	H
CZ R572	1	0.77	H
CD E578	1	0.82	N/A
CD E583	2	0.84	H
CD E589	2	0.33	T

a) atom depth indexes obtained with a probing sphere of 1.2 nm radius; b) DSSP nomenclature: H = α-helix, B = in isolated β-bridge, E = extended strand, G = 3_{10} helix, T = hydrogen bonded turn, S = bend.
